# Exploring consequences of simulation design for apparent performance of methods of meta-analysis

**DOI:** 10.1177/09622802211013065

**Published:** 2021-06-10

**Authors:** Elena Kulinskaya, David C. Hoaglin, Ilyas Bakbergenuly

**Affiliations:** 1 School of Computing Sciences, University of East Anglia, Norwich, UK; 2 Department of Population and Quantitative Health Sciences, University of Massachusetts Medical School, Worcester, MA, USA

**Keywords:** Meta-analysis, odds-ratio, random-effects model, random probabilities, random sample sizes

## Abstract

Contemporary statistical publications rely on simulation to evaluate performance of new methods and compare them with established methods. In the context of random-effects meta-analysis of log-odds-ratios, we investigate how choices in generating data affect such conclusions. The choices we study include the overall log-odds-ratio, the distribution of probabilities in the control arm, and the distribution of study-level sample sizes. We retain the customary normal distribution of study-level effects. To examine the impact of the components of simulations, we assess the performance of the best available inverse–variance–weighted two-stage method, a two-stage method with constant sample-size-based weights, and two generalized linear mixed models. The results show no important differences between fixed and random sample sizes. In contrast, we found differences among data-generation models in estimation of heterogeneity variance and overall log-odds-ratio. This sensitivity to design poses challenges for use of simulation in choosing methods of meta-analysis.

## 1 Introduction

Many methodological publications in applied statistics develop a new method, illustrate it in examples, and evaluate its performance by simulation. Our interest lies in methods for meta-analysis (MA). For meta-analysis of odds ratios, we demonstrate how researchers’ choices of simulation design can affect conclusions on the comparative merits of various methods.

Presentations of meta-analysis methods usually include assumptions about the behavior of the estimates from the individual studies. For example, a generic 2-stage random-effects model relates the observed effect sizes *y_i_* (*i *=* *1, …, *K*) to the overall effect *μ* in the model

(1) $$y_{i}=\mu +\delta_{i}+\varepsilon_{i}$$

where $\delta_{i}∼N(0,\tau^{2})$ represent random variation in the underlying study-level effects, the $\varepsilon_{i}∼N(0,\sigma_{i}^{2})$ represent random variation within the studies, and the *δ_i_* and the $\varepsilon_{i}$ are independent. From the *y_i_* and their estimated variances, $s_{i}^{2}={\hat{\sigma }}_{i}^{2}$, the two-stage method estimates *μ* and also $\tau^{2}$. Such a model can serve as a basis for analysis and also as the basis for generating data as part of a simulation study. The analysis model and the data-generation model may differ, however. For example, when the measure of effect is the log-odds-ratio, the data-generation model produces more-basic study-level data (such as numbers of events in the two arms, as shown in Section 2), from which *y_i_* and $s_{i}^{2}$ are calculated, and the popular inverse–variance–weighted methods build on equation (1). On the other hand, other methods, such as generalized linear models, build on the likelihood for the distributions in the data-generation model. In order to study the impact of choices among data-generation models – our primary interest – our simulations use several analysis models and methods based on them.

For a particular method, one can regard a measure of performance, such as the bias of a point estimator or the coverage of an interval estimator, as a function of variables that describe the meta-analysis and its setting. By a combination of analysis and, mainly, simulation, one aims to evaluate that function and describe its behavior. The variables include the number of studies, the study-level sample sizes, the extent of imbalance of the arm-level sample sizes, the overall effect, the between-study variance of the effect (for a random-effects method), and the arm-level variances within the studies (if the effect is continuous); and the relation of the performance measure to the variables usually involves nonlinearities and interactions. Thus, the design of a simulation has important implications for accuracy in evaluating the function, for estimating those relations, and, especially, for relevance of the results to practice.

The conventional meta-analysis of odds ratios from *K* studies involves 2*K* binomial variables, $X_{ij}∼\text{Bin}(n_{ij},p_{ij})$ for i=1,…,K and *j *=* C* or *T* (for the Control or Treatment arm). The random-effects model assumes that $\text{logit}(p_{ij})=\alpha_{i}+\theta_{i}z_{ij}$ for $\theta_{i}∼N(\theta ,\tau^{2})$ and an indicator *z_ij_* taking on values 0 (for Control) and 1 (for Treatment). In this notation, $\alpha_{i}=\text{logit}(p_{iC})$ and $\alpha_{i}+\theta_{i}=\text{logit}(p_{iT})$.

A design specifies a systematic collection of situations involving the number of studies, *K*; the sample sizes, *n_ij_*; the control-arm probabilities, *p_iC_*, or, equivalently, their logits, *α_i_*; the overall log-odds-ratio, *θ*; and the between-study variance, $\tau^{2}$. For each situation the simulation uses *M* replications, where *M* is typically large, say 10,000.

For simplicity, we consider equal arm-level sample sizes, $n_{iC}=n_{iT}=n_{i}$; some studies use a random allocation ratio centered at a given percentage, *q*. Studies vary in how they specify the *n_i_*. Choices include setting $n_{1}=\cdots =n_{K}$ with the same value in all *M* replications, using a fixed set of *n_i_* (not all equal), and using some distribution (typically normal or uniform) to generate a new set of *n_i_* in each replication.

Similarly, the *p_iC_* or their logits *α_i_* can be fixed or generated from some distribution. Again, normal and uniform distributions are the typical choices.

For $\tau^{2}$ most studies use a few selected values or an equally spaced set, such as $\tau^{2}$ = 0, 0.1, …, 1, though some generate $\tau^{2}$ randomly.^1^ Some studies specify values of the heterogeneity measure *I*^2^ and obtain values of $\tau^{2}$ indirectly.

In Section 2, we review approaches for generating log-odds-ratios and control-arm probabilities, and consider their statistical consequences. For two-stage methods of meta-analysis, which use the studies’ sample log-odds-ratios and their estimated variances, the relation between the estimates and their inverse-variance weights can produce bias. Section 3 examines this complication analytically, for fixed study-level sample sizes. Section 4 discusses approaches for generating sample sizes randomly and analyzes their impact. In Section 5 we study, by simulation, how various choices in generating data affect comparative merits of several established meta-analytic methods in estimating the between-study variance $\tau^{2}$ and the overall log-odds-ratio *θ*. The methods we study include the best available two-stage methods for MA: the Mandel-Paule estimator of $\tau^{2}$ and the corresponding inverse–variance–weighted estimator of *θ* with a confidence interval based on the normal distribution. We also consider the performance of two GLMM methods and a two-stage estimator of *θ* with constant sample-size-based weights whose confidence interval is based on the *t* distribution. Section 6 describes and summarizes the results. Discussion, in Section 7, offers concluding remarks. Appendices 1 and 2 provide technical details for Section 3. Additional figures are provided in online Supplemental material.

## 2 Generation of log-odds-ratios and control-arm probabilities

Consider *K* studies that used a particular individual-level binary outcome. Each study reports *X_iT_* and *X_iC_*, the numbers of events in the *n_iT_* subjects in the Treatment arm and the *n_iC_* subjects in the Control arm, for i=1,…,K. It is customary to treat *X_iT_* and *X_iC_* as independent binomial variables

(2) $$X_{iT}∼\text{Bin}(n_{iT},\;p_{iT})\;\text{ and }\;X_{iC}∼\text{Bin}(n_{iC},\;p_{iC})$$

The log-odds-ratio for Study *i* is

(3) $$\theta_{i}=\log_{e}\left(\frac{p_{iT}(1-p_{iC})}{p_{iC}(1-p_{iT})}\right)\;\text{ estimated}\;\text{by }\;{\hat{\theta }}_{i}=\log_{e}\left(\frac{{\hat{p}}_{iT}(1-{\hat{p}}_{iC})}{{\hat{p}}_{iC}(1-{\hat{p}}_{iT})}\right)$$

The (conditional, given *p_ij_* and *n_ij_*) variance of ${\hat{\theta }}_{i}$, derived by the delta method, is

(4) $$v_{i}^{2}=\text{Var}({\hat{\theta }}_{i})=\frac{1}{n_{iT}p_{iT}(1-p_{iT})}+\frac{1}{n_{iC}p_{iC}(1-p_{iC})}$$

estimated by substituting ${\hat{p}}_{ij}$ for *p_ij_*. (In analyses, we follow the particular method’s procedure for calculating ${\hat{p}}_{ij}$.)

Under the binomial-normal random-effects model (REM), the true study-level effects, *θ_i_*, follow a normal distribution

(5) $$\theta_{i}∼N(\theta ,\tau^{2})$$

For analysis, the resulting logistic mixed-effects model belongs to the class of generalized linear mixed models (GLMMs).^2,3^ Kuss,^1^ Jackson et al.,^4^ and Bakbergenuly and Kulinskaya^5^ review these GLMM methods.

In practice, *p_iC_* and *p_iT_* vary among studies in a variety of ways, not necessarily described by any particular distribution. Almost all analyses and simulations use the binomial-normal REM for the relation between *p_iT_* and *p_iC_*. Simulations can treat the *p_iC_* as constant (e.g. at a sequence of values) or sample them from a distribution, either directly (usually from a uniform distribution or a more general beta distribution; Section 2.2 discusses beta and beta-binomial models) or indirectly, by generating $\text{logit}(p_{iC})$ (usually from a Gaussian distribution).

For reference, Table 1 lists the various data-generation models considered in more detail later.

**Table 1.** Summary of data-generation models for log-odds-ratio.

Data-		Study-level	Fraction of
generation	Intercept	random	random effect
model	*α_i_* or $\alpha +u_{i}$	effects *b_i_*	in Control arm (*c*)
FIM1	fixed *α_i_*	$N(0,\tau^{2})$	0
FIM2	fixed *α_i_*	$N(0,\tau^{2})$	1/2
RIM1	$u_{i}∼N(0,\sigma^{2})$	$N(0,\tau^{2})$	0
RIM2	$u_{i}∼N(0,\sigma^{2})$	$N(0,\tau^{2})$	1/2
URIM1	*p_iC_* uniform	$N(0,\tau^{2})$	0
FIM1F	fixed *α_i_*	$\tau^{2}=0$	N/A
RIM1F	$u_{i}∼N(0,\sigma^{2})$	$\tau^{2}=0$	N/A
URIM1F	*p_iC_* uniform	$\tau^{2}=0$	N/A

Note: In the fixed-intercept models, $\log(p_{iT}/(1-p_{iT}))=\alpha_{i}+\theta +(1-c)b_{i}$ and $\log(p_{iC}/(1-p_{iC}))=\alpha_{i}-cb_{i}$. In the random-intercept models, $\log(p_{iT}/(1-p_{iT}))=\alpha +u_{i}+\theta +(1-c)b_{i}$ and $\log(p_{iC}/(1-p_{iC}))=\alpha +u_{i}-cb_{i}$.

### 2.1 Models with fixed and random intercepts

We consider two fixed-intercepts random-effects models (FIM1 and FIM2, Section 2.1.1) and two random-intercept random-effects models (RIM1 and RIM2, Section 2.1.2) as in Bakbergenuly and Kulinskaya.^5^ These models are equivalent to Models 2 and 4 (for FIM) and Models 3 and 5 (for RIM), respectively, of Jackson et al.^4^ Briefly, the FIMs include fixed control-arm effects (log-odds of the control-arm probabilities), and the RIMs replace these fixed effects with random effects.

Under the fixed-effect (common-effect) model, $\tau^{2}=0$ and $\theta_{i}\equiv \theta$. Still, the control-arm effects can be either fixed or random, resulting in two fixed-effect models: the fixed-intercepts fixed-effect model FIM1F, and the random-intercept fixed-effect model RIM1F. Random-intercept fixed-effect models were considered by Kuss^1^ and Piaget-Rossel and Taffé.^6^ However, GLMMs with random *θ_i_* are traditional in meta-analysis.

#### 2.1.1 Fixed-intercepts models (FIM1 and FIM2)

The fixed-intercepts models assume fixed effects for the studies’ control arms and allow heterogeneity in odds ratios among studies. (We follow Rice et al.^7^ in using the plural form for fixed intercepts that differ among the studies.) Given the binomial distributions in the two arms (equation (2)), the model is (i=1,…,K)

(6) $$\begin{array}{l} \log\left(\frac{p_{iT}}{1-p_{iT}}\right)=\alpha_{i}+\theta +(1-c)b_{i}\\ \log\left(\frac{p_{iC}}{1-p_{iC}}\right)=\alpha_{i}-cb_{i} \end{array}$$

where the *α_i_* are the fixed control-arm effects, *θ* is the overall log-odds-ratio, and the $b_{i}∼N(0,\tau^{2})$ are random effects. Under FIM1, *c *=* *0, resulting in higher variance in the treatment arm. Under FIM2, *c *=* *1/2, splitting the random effect *b_i_* equally between the two equations and yielding equal variance in the two arms. When $\tau^{2}\equiv 0$, these two models become a fixed-intercepts fixed-effect model, FIM1F.

An analysis has to estimate the fixed study-specific intercepts *α_i_* (usually regarded as nuisance parameters), along with *θ* and $\tau^{2}$. In a logistic mixed-effects regression, these *K *+* *2 parameters are estimated iteratively, using marginal quasi-likelihood, penalized quasi-likelihood, or a first- or second-order-expansion approximation. Jackson et al.^4^ demonstrate that inference using FIM2 is preferable, even though they generate data from FIM1.

#### 2.1.2 Random-intercept models (RIM1 and RIM2)

As *K* becomes large, it may be inconvenient, even problematic, for analysis to have a separate *α_i_* for each study. One can replace those fixed intercepts with random intercepts $\alpha +u_{i}$, centered at *α*:

(7) $$\begin{array}{l} \log\left(\frac{p_{iT}}{1-p_{iT}}\right)=\alpha +u_{i}+\theta +(1-c)b_{i}\\ \log\left(\frac{p_{iC}}{1-p_{iC}}\right)=\alpha +u_{i}-cb_{i} \end{array}$$

As before, *θ* is the overall log-odds-ratio, and $b_{i}∼N(0,\tau^{2})$. RIM1 and RIM2 correspond to *c *=* *0 and 1/2, respectively. Now the $u_{i}∼N(0,\sigma^{2})$, and *u_i_* and *b_i_* can be correlated: $\text{Cov}(u_{i},b_{i})=\rho \sigma \tau$. (If this bivariate normal distribution is not correct, however, estimates of *θ* will be biased.^8^) Under RIM1, heterogeneity of log-odds is represented in the control arms by the variance $\sigma^{2}$ and in the treatment arms by $\sigma^{2}+2\rho \sigma \tau +\tau^{2}$. Typically, *ρ* is taken as zero in simulation. The standard two-stage random-effects analysis model, which works with the sample log-odds-ratios, involves only a single between-study variance, $\tau^{2}$. Turner et al.^2^ point out that *ρ* should be estimated. Estimation of *α*, *θ*, $\sigma^{2},\;\tau^{2}$ and *ρ* is similar to estimation of the parameters in the fixed-intercept model.^2^ Again, RIM2 is preferable to RIM1 for inference.

When $\tau^{2}\equiv 0$, these two models become a random-intercept fixed-effect model, denoted by RIM1F.

The vast majority of simulation studies use FIM1 or RIM1 for data generation, both for standard two-stage methods of MA and when studying performance of GLMMs, even when they use FIM2 or RIM2 for inference. Examples include Sidik and Jonkman,^9^ Platt et al.,^10^ Bakbergenuly and Kulinskaya,^5^ and Cheng et al.^11^ for FIM, and Abo-Zaid et al.^12^ (σ=0.25 and 1.5), Kosmidis et al.^13^ ($\sigma^{2}=0.1$), and Jackson et al.^4^ (Settings 1 to 12) (σ=0.3) for RIM.

Langan et al.^14^ use a somewhat more complicated simulation scheme, which either fixes the average within-study probabilities ${\bar{p}}_{i}$ (at .5, .05, and .001) or generates them from U(.1,.5), and then derives the values of *p_iC_* and *p_iT_* from the values of ${\bar{p}}_{i}$ and *θ_i_*, the latter normally distributed as in equation (5). Thus, *p_iC_* satisfies the equation $\text{logit}(p_{iC})=\text{logit}(2{\bar{p}}_{i}-p_{iC})-\theta_{i}$. So $\text{logit}(p_{iC})$ has a share of the variance, making this a version of FIM2 or RIM2.

#### 2.1.3 Moments of the control-arm probability under RIM

The Gaussian random-intercept models generate the control-arm probabilities, *p_iC_*, indirectly: $\text{logit}(p_{iC})$ has a normal distribution centered at $\alpha =\text{logit}(p_{C}^{0})$. On the probability scale, where $p_{C}^{0}=\text{expit}(\alpha )=\exp(\alpha )/(1+\exp(\alpha ))$, the distribution is unimodal and skewed to the right when $p_{C}^{0}<0.5$. Thus, simulations from RIM involve, on average, higher control-arm probabilities than corresponding simulations from FIM, though the median control-arm probability is the same. (In FIM1, the distribution has a single value: $p_{iC}=\text{expit}(\alpha_{i})$.) To aid in comparing FIM and RIM, we evaluate the mean and variance of this distribution; we use the standard delta method.

For a transformed random variable Y=h(X)

(8) $$\text{E}(Y)=h(\text{E}(X))+h^{\prime \prime }(\text{E}(X))\text{Var}(X)/2\;\text{ and }\;\text{Var}(Y)={\left[h^{\prime }(\text{E}(X))\right]}^{2}\text{Var}(X)$$

For $\alpha_{i}=\text{E}(\text{logit}(p_{iC}))$ and $p_{C}^{0}=\text{expit}(\alpha )$, we have

$$p_{C}^{0}=h(\alpha )=\frac{\exp(\alpha )}{1+\exp(\alpha )}=1-\frac{1}{1+\exp(\alpha )}$$

The derivatives of h(·) at *α* are

$$h^{\prime }(\alpha )=\frac{\exp(\alpha )}{{\left(1+\exp(\alpha )\right)}^{2}}=p_{C}^{0}(1-p_{C}^{0})$$

and

$$h^{\prime \prime }(\alpha )=\frac{\exp(\alpha )(1-\exp(\alpha ))}{{\left(1+\exp(\alpha )\right)}^{3}}=p_{C}^{0}(1-p_{C}^{0})(1-2p_{C}^{0})$$

Hence

$$\text{E}(p_{iC})=p_{C}^{0}+p_{C}^{0}(1-p_{C}^{0})(1-2p_{C}^{0})\sigma^{2}/2\text{ and Var}(p_{iC})={\left[p_{C}^{0}(1-p_{C}^{0})\right]}^{2}\sigma^{2}$$

The mean probability increases with the variance, $\sigma^{2}$, of the normal distribution of *u_i_*. For $p_{C}^{0}=.1$, say, the mean is .100 when $\sigma^{2}=0.01$, but it increases to .109 for $\sigma^{2}=0.25$ and to .136 for $\sigma^{2}=1$. Therefore, simulations from FIM and RIM are not quite equivalent.

### 2.2 Non-Gaussian random-intercept models

Other distributions besides the Gaussian can serve as a mixing distribution for control-arm probabilities.

In Bayesian analysis, the beta distributions are conjugate priors for a binomial, so they are a natural choice for a mixing distribution. The result is a marginal beta-binomial distribution in the control arm. In meta-analysis a beta-binomial model^1,15^ usually assumes beta-binomial distributions in both arms. However, Bakbergenuly and Kulinskaya^15^ showed that the standard RE method does not perform well when the data come from a beta-binomial model. Therefore, we would not use a RIM with beta-generated probabilities.

We are not aware of any simulation studies that intentionally used a beta distribution for control-arm probabilities. However, the Beta(1,1) distribution is the same as U(0, 1), and a popular choice is a uniform distribution on an interval, $\left(p_{l},\;p_{u})\subset [0,1\right]$. Viechtbauer,^16^ Sidik and Jonkman,^17^ and Nagashima et al.^18^ (Set iii) generated the *p_iC_* from U(.05, .65) in combination with the Gaussian REM. Similarly, Jackson et al.^4^ (Setting 13) generated the *p_iC_* from U(.1, .3). All these studies add a uniform distribution of control-arm probabilities to the FIM1 setting, producing a random-intercept model that we denote by URIM1. This model retains the normal distribution of the *θ_i_*.

Piaget-Rossel and Taffé^6^ used a fixed-effect model with $p_{iC}∼U(p-p/5,\;p+p/5)$, URIM1F in our nomenclature, with p=.1,.007,.0035,.0015. Piaget-Rossel^19^ used the same distribution for the *p_iC_* and uniformly distributed log-odds-ratios, $\theta_{i}∼U(\theta \pm \sqrt{3\tau^{2}})$.

If X∼Bin(n,p) and p∼U(0,1), then *X* has the discrete uniform distribution U(0,1,…,n). More generally, when $p∼U(p_{l},\;p_{u})$, the probabilities for the numbers of successes are

(9) $$\begin{array}{c} P(X=k)=\frac{1}{p_{u}-p_{l}}\int_{p_{l}}^{p_{u}}\left(\begin{array}{c} n\\ k \end{array}\right)u^{k}(1-u{)}^{n-k}\text{d}u\\ =\frac{1}{p_{u}-p_{l}}[B(p_{u};k+1,n-k+1)\\ \;-B(p_{l};k+1,n-k+1)] \end{array}$$

where B(·;·,·) denotes the incomplete beta function. To examine the effects on the performance of the MA methods, our simulations include uniform distributions of control-arm probabilities.

## 3 Variances and covariances of estimated log-odds-ratios and their weights

Traditional one-size-fits-all meta-analysis proceeds in two stages: obtain the study-level estimates and their estimated variances (or standard errors) and then estimate the overall effect as a weighted mean with inverse-variance weights. One of its main faults is that it ignores the variability of the estimated variances. As a result, the variance of the overall effect is underestimated.^20^ Additionally, a relation between the estimated study-level effects and their estimated variances may lead to bias in the estimate of the overall effect. In this section, we explore these relations for the log-odds-ratio and its variance and inverse-variance weight. We also demonstrate that the relation varies with the data-generation mechanism. In particular, the sample log-odds-ratio and its estimated variance can be almost independent under FIM2 and RIM2 when *θ* = 0. Because the calculations are somewhat easier, we first examine the relation to the estimated variance and then turn to the relation to the inverse-variance weight.

### 3.1 Relation of sample log-odds-ratio and its estimated variance

The data-generation mechanisms for the random-effects model generate the *p_iC_* and *p_iT_* and then generate *X_iC_* and *X_iT_*, according to equation (2). Thus, to obtain the covariance between ${\hat{\theta }}_{i}$ and $\hat{\text{Var}}({\hat{\theta }}_{i})$, we apply the law of total covariance

(10) $$\text{Cov}({\hat{\theta }}_{i},\hat{\text{Var}}({\hat{\theta }}_{i}))=\text{E}[\text{Cov}({\hat{\theta }}_{i},\hat{\text{Var}}({\hat{\theta }}_{i})|\alpha_{i},\;\theta_{i})]+\text{Cov}(\text{E}({\hat{\theta }}_{i}|\alpha_{i},\;\theta_{i}),\text{E}(\hat{\text{Var}}({\hat{\theta }}_{i})|\alpha_{i},\;\theta_{i}))$$

In the process, to show the full effect of the data-generating mechanism, we also obtain $\text{Var}({\hat{\theta }}_{i})$, using the more-familiar law of total variance

(11) $$\text{Var}({\hat{\theta }}_{i})=\text{E}[\text{Var}({\hat{\theta }}_{i}|\alpha_{i},\;\theta_{i})]+\text{Var}(\text{E}({\hat{\theta }}_{i}|\alpha_{i},\;\theta_{i}))=\text{E}(v_{i}^{2})+\tau^{2}$$

In equation (10), the covariance of the conditional expectations is just $\text{Cov}(\theta_{i},v_{i}^{2})$ because $\text{E}({\hat{\theta }}_{i}|\alpha_{i},\;\theta_{i})=\theta_{i}=\theta +b_{i}$ and (to first order) $\text{E}(\hat{\text{Var}}({\hat{\theta }}_{i})|\alpha_{i},\;\theta_{i})=v_{i}^{2}$. Thus, we need to calculate $\text{Cov}({\hat{\theta }}_{i},\hat{\text{Var}}({\hat{\theta }}_{i})|\alpha_{i},\;\theta_{i})$ and take its expectation. Conditioning on *α_i_* and *θ_i_*, in equation (6) and equation (7), is equivalent to conditioning on *p_iC_* and *p_iT_*. Therefore, we can rewrite equation (10) as (shortening $\hat{\text{Var}}({\hat{\theta }}_{i})$ to ${\hat{v}}_{i}^{2}$)

$$\text{Cov}({\hat{\theta }}_{i},{\hat{v}}_{i}^{2})=\text{E}[\text{Cov}({\hat{\theta }}_{i},{\hat{v}}_{i}^{2}|p_{iC},\;p_{iT})]+\text{Cov}(\theta_{i},v_{i}^{2})$$

The first term in the above equation accounts for the binomial variation (of order $1/n_{i}$) in ${\hat{\theta }}_{i}$ and in ${\hat{v}}_{i}^{2}$, given *p_iC_* and *p_iT_*, whereas the second term accounts for the variation of its expected value and variance from random effects, of order 1 in model (7). Therefore, the first, binomial term is of smaller order ($O(n_{i}^{-2})$) than the second term (the covariance of the expected moments) and can be neglected in a calculation to order $1/n_{i}$.

To calculate the covariance of *θ_i_* and $v_{i}^{2}$, we assume, for simplicity, that *u_i_* and *b_i_* are independent in RIM1 and RIM2. Then, defining $p_{C}=\text{expit}(\alpha )$ and $p_{T}=\text{expit}(\alpha +\theta )$, to order $1/n_{i}$

(12) $$\text{Cov}(\theta_{i},v_{i}^{2})=\frac{c\tau^{2}}{n_{iC}}\left[\frac{1-2p_{C}}{p_{C}(1-p_{C})}\right]-\frac{(1-c)\tau^{2}}{n_{iT}}\left[\frac{1-2p_{T}}{p_{T}(1-p_{T})}\right]$$

In particular, when *c *=* *1/2, *θ* = 0 and *n_iT_* = *n_iC_*, $\text{Cov}(\theta_{i},v_{i}^{2})=0$.

After some algebra, we also obtain, to order $1/n_{i}$,

(13) $$\begin{array}{l} \text{Var}({\hat{\theta }}_{i})={\left[n_{iT}p_{T}(1-p_{T})\right]}^{-1}+[n_{iC}p_{C}(1-p_{C}){]}^{-1}+\tau^{2}\\ \;+\left(\sigma^{2}+{\left(1-c\right)}^{2}\tau^{2}+2(1-c)\rho \sigma \tau \right)[2n_{iT}{]}^{-1}\left({\left[p_{T}(1-p_{T})\right]}^{-1}-2\right)\\ \;+\left(\sigma^{2}+c^{2}\tau^{2}-2c\rho \sigma \tau \right)[2n_{iC}{]}^{-1}\left({\left[p_{C}(1-p_{C})\right]}^{-1}-2\right) \end{array}$$

The binomial variance component $v_{i}^{2}$ is inflated by allowing random effects/random intercepts. The extent of the inflation involves $\tau^{2},\;\sigma^{2}$, and *c*.

Appendix 1 shows derivations for equations (12) and (13), and Appendix 2 applies those results to an arbitrary distribution of *p_C_*, including URIM1 as a special case.

### 3.2 Relation of sample log-odds-ratio and its weight

We can write the IV weights as ${\hat{w}}_{i}={\hat{v}}_{i}^{-2}/({\hat{W}}_{\left(i\right)}+{\hat{v}}_{i}^{-2})={\left[{\hat{W}}_{\left(i\right)}{\hat{v}}_{i}^{2}+1\right]}^{-1}$, where W^(i)=∑j=iv^j−2 is independent of ${\hat{v}}_{i}^{2}$ and of ${\hat{\theta }}_{i}$. Similarly, let W(i)=∑j=ivj−2. We are interested in $\text{Cov}({\hat{\theta }}_{i},{\hat{w}}_{i})$. Again using the law of total covariance

$$\text{Cov}({\hat{\theta }}_{i},{\hat{w}}_{i})=\text{E}[\text{Cov}({\hat{\theta }}_{i},{\hat{w}}_{i}|\alpha_{i},\;\theta_{i})]+\text{Cov}(\theta_{i},w_{i}^{0})$$

where $w_{i}^{0}=\text{E}({\hat{w}}_{i}|\alpha_{i},\;\theta_{i})={\left[W_{\left(i\right)}v_{i}^{2}+1\right]}^{-1}+O(1/n_{i})$. The first term of the covariance is of a smaller order than the second, so to order 1/n, $\text{Cov}({\hat{\theta }}_{i},{\hat{w}}_{i})=\text{Cov}(\theta_{i},{\left[W_{\left(i\right)}v_{i}^{2}+1\right]}^{-1})$.

Expanding ${\left[W_{\left(i\right)}v_{i}^{2}+1\right]}^{-1}$ and taking into account the independence of $W_{\left(i\right)}$ from *θ_i_* and $v_{i}^{2}$, we have

(14) $$\text{Cov}(\theta_{i},{\left[W_{\left(i\right)}v_{i}^{2}+1\right]}^{-1})=-\frac{\text{E}(W_{\left(i\right)})}{{\left(\text{E}(W_{\left(i\right)})\text{E}(v_{i}^{2})+1\right)}^{2}}\text{Cov}(\theta_{i},v_{i}^{2})$$

where E(W(i))=∑j=iE(vj−2).

Equations (12) to (14) show that the choice of *θ*, the choice of *p_C_* (through *α*), the choice of FIM versus RIM (through $\sigma^{2}$), the choice of fixed-effect versus random-effects model (through $\tau^{2}$), and the choice of FIM1/RIM1 versus FIM2/RIM2 (through *c*) all affect the covariances between ${\hat{\theta }}_{i}$ and their estimated weights, and result in varying biases in the estimated overall effect. In particular, when $n_{T}=n_{C},\;\theta =0$, and *c *=* *1/2, the covariance is zero, so the ${\hat{\theta }}_{i}$ and their estimated weights are almost independent, making the standard IV estimate of the overall effect unbiased when generated from FIM2/RIM2. On the other hand, the sign of the bias of the ${\hat{\theta }}_{i}$ depends on the sign of $1-2p_{T}$, and the bias increases with an increase in $\tau^{2}$ when generated from FIM1/RIM1.

## 4 Generation of sample sizes

Several authors^5,11^ use constant study-level sample sizes, either equal or unequal, in all replications. More often, however, authors generate sample sizes from a uniform or normal distribution. Jackson et al.^4^ use (mostly with *n_iC_* = *n_iT_*) sample sizes from discrete *U*(50, 500). Langan et al.^14^ use either constant and equal sample sizes within and across studies, or sample sizes from U(40, 400) and U(2000, 4000); Sidik and Jonkman^17^ use U(20, 200), and Abo-Zaid et al.^12^ use U(30, 100) and U(30, 1000). Viechtbauer^16^ generates study-level sample sizes ($n_{i}=n_{iC}=n_{iT}$) from N(n, n/4) (n/4 is the variance) with n=10,20,40,80,160. In an extensive simulation study for sparse data, Kuss^1^ uses FIM1F and FIM1 along with a large number of fitting methods. He generates both the number of studies *K* and their sample sizes *n* from log-normal distributions: with mean 0.65 and standard deviation 1.2 for rather small *K*, with log-normal mean 3.05 and log-normal standard deviation 0.97 for larger *K*, and with log-normal mean 4.615 and log-normal standard deviation 1.1 for sample sizes. He applies the ceiling function to the generated number and adds 1, and he limits the number of studies to a maximum of 100.

In general, if mutually independent random variables *Y_i_* have a common distribution F(·), and $N∼G_{n}(\cdot )$ is independent of the *Y_i_*, the sum $Y_{1}+\cdots +Y_{N}$ has a *c*ompound distribution.^21^ A binomial distribution with probability *p* and a random number of trials is a compound Bernoulli distribution. The first two moments of such a distribution are E(X)=pE(N) and $\text{Var}(X)=p(1-p)\text{E}(N)+p^{2}\text{Var}(N)$. This variance is larger than the variance of the Bin(E(N), p) distribution. Therefore, random generation of sample sizes produces an overdispersed binomial (compound Bernoulli) distribution for the control arm, and may also inflate, though in a more complicated way, the variance in the treatment arm.

In particular, when $N∼N(\text{E}(N),\sigma_{n}^{2})$, the variance $\text{Var}(X)=p(1-p)\text{E}(N)+p^{2}\sigma_{n}^{2}$. And when $N∼U(n_{l},\;n_{u}),\;\text{Var}(X)=p(1-p)\text{E}(N)+p^{2}{\left(n_{u}-n_{l}\right)}^{2}/12$.

### 4.1 Variances and covariances of estimated log-odds-ratios and their weights for random sample sizes

To calculate the variance of $\hat{\theta }$ when sample sizes are random, we again use the law of total variance

$$\text{Var}({\hat{\theta }}_{i})=\text{E}(\text{Var}({\hat{\theta }}_{i}|n_{i}))+\text{Var}(\text{E}({\hat{\theta }}_{i}|n_{i}))$$

The second term is Var(θ)=0, and the first term is obtained by substituting $\text{E}(n_{iC}^{-1})$ and $\text{E}(n_{iT}^{-1})$ in equation (13).

Using the delta method

(15) $$\text{E}(N^{-1})={\left(\text{E}(N)\right)}^{-1}(1+{\left[\text{CV}(N)\right]}^{2})$$

where the coefficient of variation, CV, is the ratio of the standard deviation of *N* to its mean. Therefore, to order 1/E(N), random generation of sample sizes inflates the variance of $\hat{\theta }$ if and only if the coefficient of variation of the distribution of sample sizes is of order 1. In the simulations of Viechtbauer,^16^ where $\text{Var}(N)=n/4,\;\text{CV}(N)=O(1/\sqrt{n})$, so the variance is not inflated. In contrast, generating sample sizes from $N(n,n^{2}/4)$ would result in CV=1/2 and would inflate variance. (Use of such a combination of mean and variance, however, is unlikely to produce realistic sets of sample sizes, and the probability of generating a negative sample size exceeds 2%.)

The variance of a uniform distribution on an interval of width Δ centered at *n*_0_ is $\Delta^{2}/12$, and its CV is $\Delta /(\sqrt{12}n_{0})$. Therefore, CV(N) is of order 1 whenever the width of the interval is of the same order as its center. Hence, variance is inflated in the simulations by Jackson,^4^ Langan et al.,^14^ Sidik and Jonkman,^17^ and Abo-Zaid et al.,^12^ who all use wide intervals for *n*.

Similarly, we use the law of total covariance to calculate the covariance between ${\hat{\theta }}_{i}$ and ${\hat{v}}_{i}^{2}$

$$\text{Cov}({\hat{\theta }}_{i},{\hat{v}}_{i}^{2})=\text{E}[\text{Cov}({\hat{\theta }}_{i},{\hat{v}}_{i}^{2}|n_{i})]+\text{Cov}(\text{E}({\hat{\theta }}_{i}),v_{i}^{2}|n_{i})$$

The second term is zero, as $\text{E}({\hat{\theta }}_{i}|n_{i})=\theta$, which does not depend on *n_i_*. So $\text{Cov}({\hat{\theta }}_{i},{\hat{v}}_{i}^{2})$ is obtained by substituting $\text{E}(n_{iC}^{-1})$ and $\text{E}(n_{iT}^{-1})$ in equation (12), and the covariances are affected only when CV(N) is of order 1.

## 5 Design of simulations

Our simulations keep the arm-level sample sizes equal in the *K* (= 5, 10, 30) studies. The control-arm probability $p_{iC}=.1,\;.4$. For the log-odds-ratios *θ_i_*, we use equation (5) with *θ* = 0, 0.5, 1, 1.5, and 2 and $\tau^{2}$ = 0, 0.1, …, 1. We vary two components of the data-generating mechanism: the model (at five levels: FIM1, FIM2, RIM1, RIM2, and URIM1) and the arm-level sample sizes, *n*, centered at 40, 100, 250, and 1000 (constant, normally distributed, or uniformly distributed). We also vary the variance $\sigma^{2}=0.1,\;0.4$ for RIM.

We keep the control-arm probabilities *p_iC_* and the log-odds-ratios *θ_i_* independent (i.e. *ρ* = 0 in the RIMs).

To make the normal and uniform distributions of sample sizes comparable, we center them at the same value *n* and equate their variances. If a normal distribution has variance $\sigma_{n}^{2}$, a uniform distribution with the same variance has interval width $\Delta_{n}=\sqrt{12\sigma_{n}^{2}}$. We set $\Delta_{n}=1.1n$, resulting in $\text{CV}=\Delta_{n}/(\sqrt{12}n)=0.318$ and a squared CV of 0.101. Therefore, by equation (15), our simulations with random *n* inflate variances and covariances by 10% in comparison with simulations with fixed *n*. Wider intervals of *n* would inflate variances more, but in generating sample sizes from a corresponding normal distribution, we want negative sample sizes to have reasonably small probability. For our choice of Δ*_n_* this probability is .0008. Unfortunately, we were still getting a small number of values below zero out of thousands of simulated values, so we additionally truncate the *n* values generated from a normal distribution at 10. Truncation happens with probability .009.

Similarly, for control-arm probabilities, even though using a normal distribution on the logit shifts the mean value of the control-arm probability, as discussed in Section 2.1.3, we can have equal variances on the probability scale by taking $\Delta_{p}=\sqrt{12{\left[p_{C}^{0}(1-p_{C}^{0})\right]}^{2}\sigma_{p}^{2}}$ in comparator simulations.

For each generated dataset, we use a number of the two-stage methods for log-odds-ratio, including the best available method:^22,23^ MP estimation of $\tau^{2}$ with corresponding inverse–variance–weighted estimation of *θ* and a confidence interval based on the normal distribution. We also consider the performance of the GLMM methods based on FIM2 and RIM2 as implemented in **metafor**.^4,5,24^ Finally, we include a weighted-average estimator of *θ* whose weights depend only on the studies’ arm-level sample sizes: $w_{i}=n_{iT}n_{iC}/(n_{iT}+n_{ic})$.^22^ We refer to this sample-size-weighted estimator as SSW. The accompanying confidence interval is based on the *t* distribution with K−1 degrees of freedom. Table 2 lists the analysis methods.

**Table 2.** Summary of methods for meta-analysis of log-odds-ratios in our simulations.

Method	Features
Two-stage methods	
Inverse–variance–weighted average	
DerSimonian-Laird (DL) estimate of $\tau^{2}$	All three assume ${\hat{v}}_{i}^{2}=v_{i}^{2}$
REML	and use
Mandel-Paule (MP) estimate of $\tau^{2}$	Normal-based CIs
	
Kulinskaya-Dollinger (KD) estimate of $\tau^{2}$	Normal-based CI
Sample-size-weighted estimator	
SSW	Constant weights
	*t*-based CI
General linear mixed models	
Binomial-normal random-effects model	
FIM2	Fixed intercept
RIM2	Random intercept

For each combination of the parameters and a data-generating mechanism, we generated data for 1000 simulated meta-analyses.

Table 3 shows the components of the simulations. For completeness we included the DerSimonian-Laird (DL), restricted maximum-likelihood (REML), MP, and Kulinskaya-Dollinger (KD) estimators of $\tau^{2}$ with the corresponding inverse–variance–weighted estimators of *θ* and confidence intervals with critical values from the normal distribution. Bakbergenuly et al.^22^ studied those inverse–variance–weighted estimators in detail. The results for the other IV-weighted estimators under the five data-generation mechanisms are similar to those for the Mandel-Paule estimator, so we do not include them in Section 6. Our preprints^25,26^ give the full details. Among the estimators, FIM2 and RIM2 denote the estimators in the corresponding GLMMs.

**Table 3.** Components of the simulations.

Parameter	Values
*K*	5, 10, 30
*n*	40, 100, 250, 1000
*θ*	0, 0.5, 1, 1.5, 2
$\tau^{2}$	0, 0.1, …, 1
*p_C_*	.1, .4
$\sigma^{2}$	0.1, 0.4

Generation of *n*	

Constant	
Normal(*n*, $1.21n^{2}/12$)	
Uniform(n±0.55n)	

Generation of $\text{logit}(p_{iC})$ and $\text{logit}(p_{iT})$	

FIM1	Section 2.1.1
FIM2	Section 2.1.1
RIM1	Section 2.1.2
RIM2	Section 2.1.2
URIM1	Section 2.2

Estimation targets	Estimators

Bias in estimating $\tau^{2}$	DL, REML, MP, KD, FIM2, RIM2
Bias in estimating *θ*	DL, REML, MP, KD, FIM2, RIM2, SSW
Coverage of *θ*	DL, REML, MP, KD, FIM2, RIM2,
	SSW (with ${\hat{\tau }}_{KD}^{2}$ and $t_{K-1}$ critical values)

## 6 Results of the simulations

In the figures that accompany the summaries of results, each plot shows a trace of a measure of the performance of an estimator (bias or coverage) for each of the five data-generation mechanisms. The horizontal variable is $\tau^{2}\in [0,1]$. A row corresponds to a value of *n* (usually 40 or 100) and a combination of values of other parameters (e.g. *p_C_* and $\sigma^{2}$ or *θ*). The figures illustrate the important patterns in the full sets of figures.^25,26^ These preprints contain full simulation results for constant, normally distributed, and uniformly distributed sample sizes *n*.

**Figure 1.**
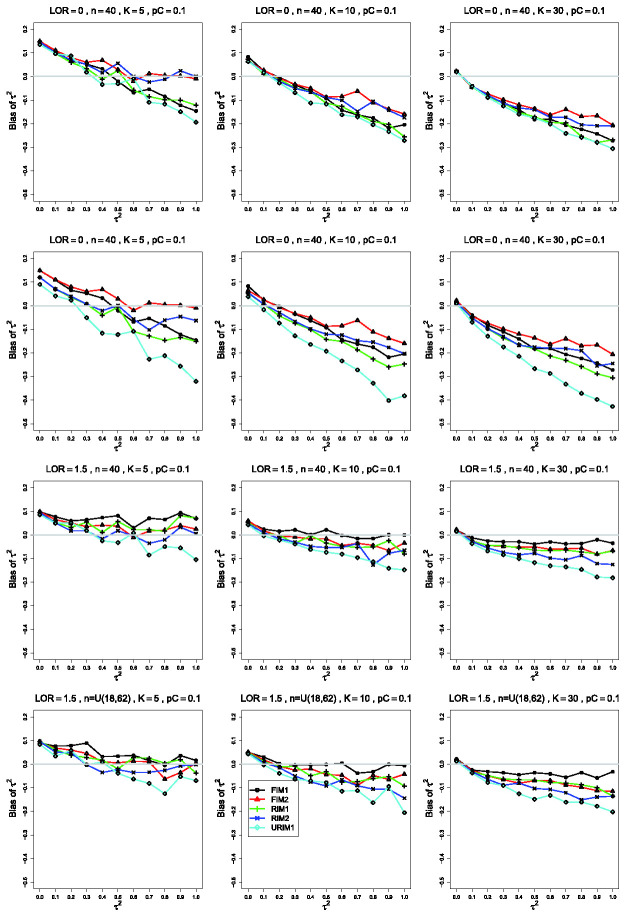
Bias in estimating the between-studies variance, $\tau^{2}$, by ${\hat{\tau }}_{MP}^{2}$ for $p_{C}=.1$, *θ* = 0, $\sigma^{2}=0.1$ (top row); *θ* = 0, $\sigma^{2}=.4$ (second row); $\theta =1.5,\;\sigma^{2}=0.4$ (bottom two rows). Sample sizes are constant *n *=* *40 in the top three rows and uniformly distributed in the bottom row. The data-generation mechanisms are FIM1 (circle), FIM2 (triangle), RIM1 (plus), RIM2 (cross), and URIM1 (diamond). Light gray line at 0.

**Figure 2.**
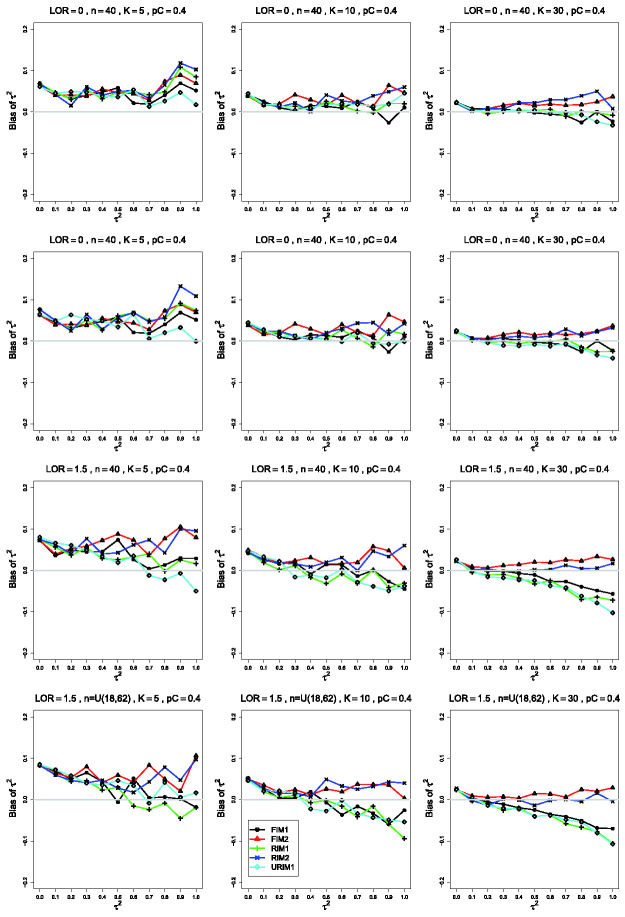
Bias in estimating the between-studies variance, $\tau^{2}$, by ${\hat{\tau }}_{MP}^{2}$ for $p_{C}=.4$, *θ* = 0, $\sigma^{2}=0.1$ (top row); *θ* = 0, $\sigma^{2}=0.4$ (second row); $\theta =1.5,\;\sigma^{2}=0.4$ (bottom two rows). Sample sizes are constant *n *=* *40 in the top three rows and uniformly distributed in the bottom row. The data-generation mechanisms are FIM1 (circle), FIM2 (triangle), RIM1 (plus), RIM2 (cross), and URIM1 (diamond). Light gray line at 0.

As it turned out, the three methods of generating sample sizes produced essentially the same results. For two illustrative examples, compare the third and fourth rows of Figures 1 and 2. Thus, with those exceptions, the plots in the figures come from the results for constant *n*.

If the five data-generation mechanisms produce the same results, their traces in a plot coincide (except for minor variation). We focus on systematic departures from this null pattern (e.g. the traces separate into two groups). The specific performance measure may be important (e.g. an estimator has substantially greater bias when the data are generated by a certain mechanism). We generally give performance less emphasis, however, because our primary goal is to examine the consequences for inference of the choice of a data-generating method. Bakbergenuly et al.^22^ have studied in detail the performance under FIM1 of the estimators other than the GLMM estimators based on FIM2 and RIM2.

### *6.1 Bias of* ${\hat{\tau }}_{MP}^{2}$

The estimated bias of ${\hat{\tau }}_{MP}^{2}$ often varies among the data-generation mechanisms. In the most common single pattern, the traces versus $\tau^{2}$ form two clusters: one for FIM2 and RIM2 and another for FIM1, RIM1, and URIM1, as shown in the first row of Figure 1. When $\sigma^{2}=0.1$ and $p_{C}=.1$, separations tend to become clearer as *K* increases, and they are most evident when *K *=* *30. As *n* increases, the traces flatten and coalesce around 0 bias, becoming essentially flat when n≥250. As *θ* increases, the traces for FIM2 and RIM2 merge with the others and then emerge below them, and the whole set of traces flattens toward 0.

Changing only *p_C_*, from .1 to .4 (Figure 2), produces traces that stay near 0. Groupings are not consistently visible. As *θ* increases, the reversal observed when $p_{C}=.1$ (particularly when *n *=* *40 and *K *=* *30) does not occur. Instead, the separation between the traces for FIM2 and RIM2 and those for FIM1, RIM1, and URIM1 increases because the latter mechanisms produce larger negative bias as $\tau^{2}$ increases.

When the simulations use $\sigma^{2}=0.4$ instead of $\sigma^{2}=0.1$, the most noticeable differences (when $p_{C}=.1$ and n≤100 and, especially, *K *=* *30) are substantially larger negative bias under URIM1 (compare the first two rows of Figure 1) and greater separation among the traces for the other mechanisms. The trace for URIM1 approaches the others as *θ* increases (compare the second and third rows of Figure 1). This change in $\sigma^{2}$ produces little change in the patterns for $p_{C}=.4$.

Turning from the data-generation mechanisms to the bias, when $p_{C}=.1$ and *θ* = 0, ${\hat{\tau }}_{MP}^{2}$ has positive bias for small to moderate values of $\tau^{2}$ and substantial negative bias when K≥10, increasing in $\tau^{2}$. FIM1, RIM1 and URIM1 produce larger negative bias than FIM2 and RIM2 when *n *=* *40. When sample sizes increase to *n *=* *100, FIM2 and RIM2 have positive bias for K≤10, whereas for *K *=* *30, FIM2 and RIM2 have almost no bias. Differences between data-generation mechanisms disappear by *n *=* *250.

Negative bias at large $\tau^{2}$ decreases with increasing *θ*. When θ≥1, *K *=* *5, and *n *=* *40, ${\hat{\tau }}_{MP}^{2}$ has a small positive bias, especially under RIM1, decreasing in *K*. For *K *=* *30, FIM1 produces almost no bias, and other mechanisms result in small negative bias. Bias is almost absent when n≥100.

When $p_{C}=.4$ and *θ* = 0, ${\hat{\tau }}_{MP}^{2}$ has a small positive bias, somewhat increasing for larger $\tau^{2}$. RIM2 and FIM2 produce somewhat more bias than the other mechanisms. When $p_{C}=.4$ and θ=1.5, FIM2 and RIM2 produce almost no bias for *K *=* *30, and the rest produce negative bias for large $\tau^{2}$. For K≤10, FIM2 and RIM2 produce positive bias, and FIM1, RIM1, and URIM1 produce positive bias for small to moderate values of $\tau^{2}$ and negative bias for large values.

### *6.2 Bias of the estimators of* $\tau^{2}$ *in the FIM2 and RIM2 GLMMs*

Having used the FIM2 and RIM2 data-generation mechanisms, we examine the performance of the estimators in those GLMMs (in this section and in Sections 6.4 and 6.7).

#### *6.2.1 Bias of* ${\hat{\tau }}_{\mathrm{FIM2}}^{2}$

**Figure 3.**
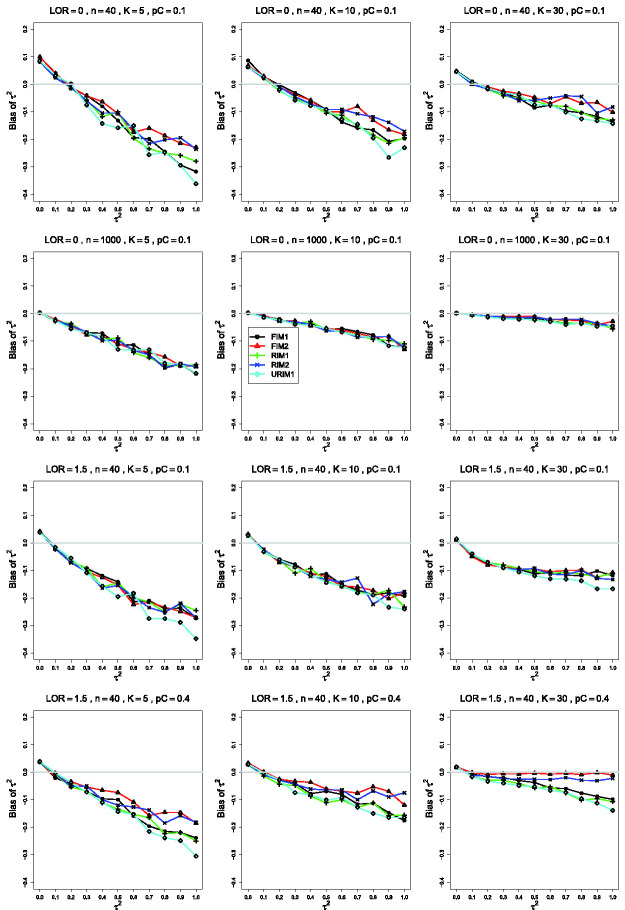
Bias of the estimator of the between-studies variance $\tau^{2}$ in the FIM2 GLMM for $\sigma^{2}=0.4,\;p_{C}=.1$, *θ* = 0 (top two rows); $p_{C}=.1,\;\theta =1.5$ (third row); $p_{C}=.4,\;\theta =1.5$ (bottom row); constant sample sizes *n *=* *40 in rows 1, 3 and 4, and *n *=* *1000 in row 2. The data-generation mechanisms are FIM1 (circle), FIM2 (triangle), RIM1 (plus), RIM2 (cross), and URIM1 (diamond). Light gray line at 0.

For the bias of ${\hat{\tau }}_{\mathrm{FIM}2}^{2}$, departures of the traces from the null pattern generally occur when *n *=* *40 and occasionally when *n *=* *100. In the most common departure, at larger $\tau^{2}$, the traces for FIM1, RIM1, and URIM1 form one group, and those for FIM2 and RIM2 form another, closer to 0, as in the first row of Figure 3. This pattern tends to become clearer as *K* increases; it occurs more often when *K *=* *30 than when *K *=* *10 or *K *=* *5.

The separation between FIM2 and RIM2 and FIM1, RIM1, and URIM1 tends to be clearer when $p_{C}=.4$ than when $p_{C}=.1$ (compare the third and fourth rows of Figure 3), and when $\sigma^{2}=0.4$ than when $\sigma^{2}=0.1$. When $p_{C}=.1$, the traces tend to be closer together as *θ* increases, but increasing *θ* has the opposite effect when $p_{C}=.4$.

In some situations, particularly when $p_{C}=.1$, *n *=* *40, *K *=* *5, and $\tau^{2}$ is larger, the trace for URIM1 is visibly lower than the other traces (as in the third row of Figure 3).

The bias of ${\hat{\tau }}_{\mathrm{FIM}2}^{2}$ under FIM2 and RIM2 relative to the other mechanisms (e.g. in the plot for *K *=* *30 in the fourth row of Figure 3) is consistent with fitting the same GLMM that generated the data and with the fact that FIM2 is a submodel of RIM2.

Except at small $\tau^{2},\;{\hat{\tau }}_{\mathrm{FIM}2}^{2}$ has negative bias, increasing with $\tau^{2}$ (as in the first row of Figure 3, where the bias exceeds –20% when $\tau^{2}=1$) but decreasing as *K* increases. The bias remains large when *θ* is larger. It is worst when *K *=* *5, even for *n *=* *1000 (second row of Figure 3). When *n *=* *40 and *K *=* *30 and $p_{C}=.4$ (but not when $p_{C}=.1$), ${\hat{\tau }}_{\mathrm{FIM}2}^{2}$ is almost unbiased under FIM2 and RIM2 (compare the third and fourth rows of Figure 3).

#### *6.2.2 Bias of* ${\hat{\tau }}_{\mathrm{RIM2}}^{2}$

**Figure 4.**
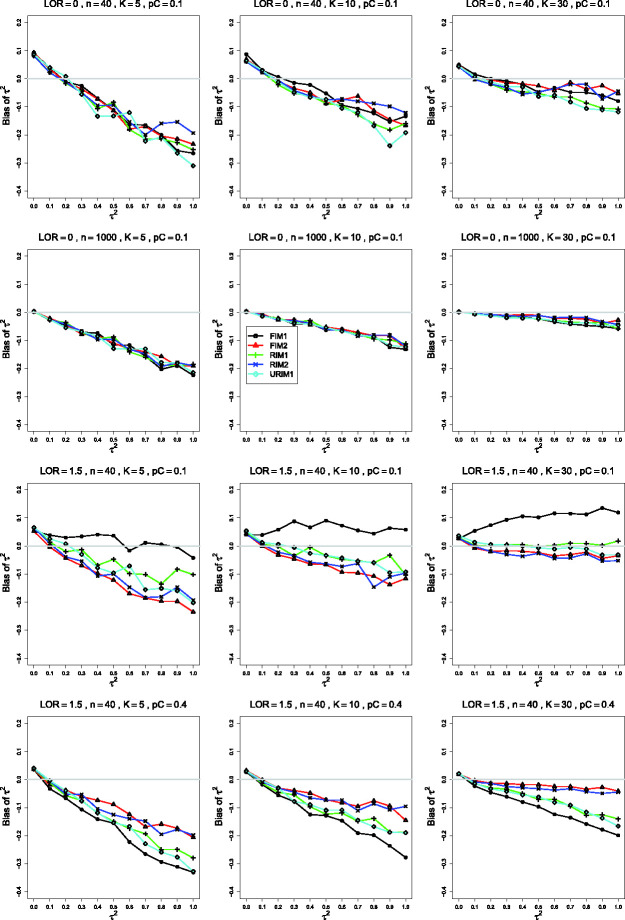
Bias of the estimator of the between-studies variance $\tau^{2}$ in the RIM2 GLMM for $\sigma^{2}=0.4,\;p_{C}=.1$, *θ* = 0 (top two rows); $p_{C}=.1,\;\theta =1.5$ (third row); $p_{C}=.4,\;\theta =1.5$ (bottom row); constant sample sizes *n *=* *40 in rows 1, 3 and 4, and *n *=* *1000 in row 2. The data-generation mechanisms are FIM1 (circle), FIM2 (triangle), RIM1 (plus), RIM2 (cross), and URIM1 (diamond). Light gray line at 0.

In summarizing the traces of the bias of ${\hat{\tau }}_{\mathrm{RIM}2}^{2}$, *p_C_* and *n* play a larger role than for ${\hat{\tau }}_{\mathrm{FIM}2}^{2}$. The pattern in which FIM2 and RIM2 form a group, above the rest (FIM1, RIM1, and URIM1), is readily evident whenever $p_{C}=.4$, and it extends to smaller $\tau^{2}$ (as in the fourth row of Figure 4). In addition to *n *=* *40 the pattern is generally present when *n *=* *100.

If $p_{C}=.1$, the same pattern is visible when $\sigma^{2}=0.1$, *θ* = 0, *K *=* *30, and *n* is 100 and 250. When *θ* is larger and *n *=* *40, however, the traces follow a different, three-group pattern: FIM1 > RIM1/URIM1 > FIM2/RIM2 (as in the third row of Figure 4).

Contrary to what one might expect, the trace for RIM2 is not always closest to 0; indeed, it is sometimes fairly far from 0, particularly when *K *<* *30 (as in the third and fourth rows of Figure 4).

Similar to ${\hat{\tau }}_{\mathrm{FIM}2}^{2},\;{\hat{\tau }}_{\mathrm{RIM}2}^{2}$ has substantial negative bias when *K *=* *5 or *K *=* *10. When *K *=* *30, ${\hat{\tau }}_{\mathrm{RIM}2}^{2}$ is nearly unbiased under RIM2 and FIM2, particularly when $p_{C}=.4$.

### *6.3 Bias of* ${\hat{\theta }}_{MP}$

The other IV-weighted estimators of *θ* have bias patterns similar to those of ${\hat{\theta }}_{MP}$.

In the traces for the bias of ${\hat{\theta }}_{MP}$, the patterns divide most clearly on *p_C_*. When $p_{C}=.1$, no plot for n≤250 shows the null pattern, whereas when $p_{C}=.4$, departures from the null pattern are rare, occurring mainly when *n *=* *40 and *K *=* *30.

**Figure 5:**
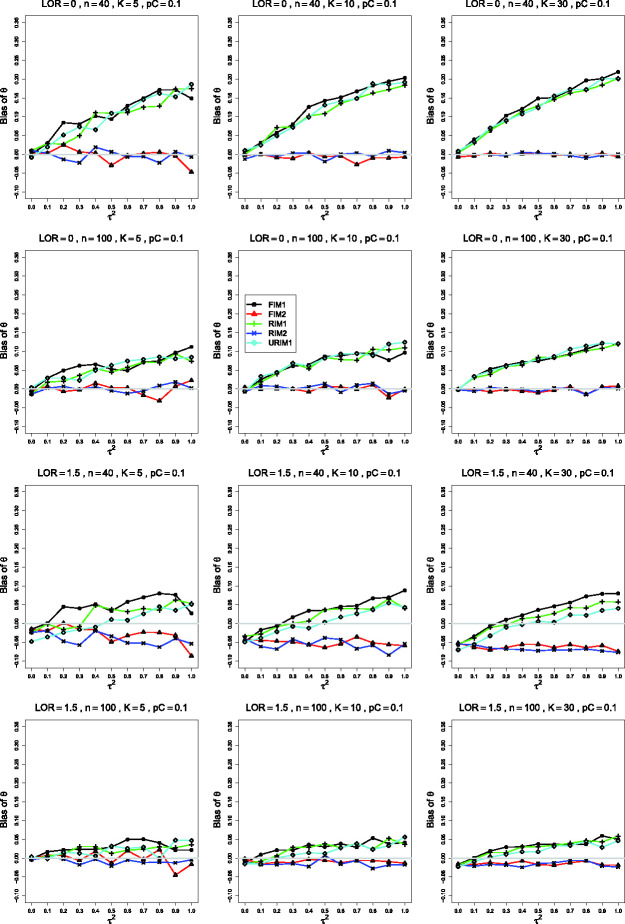
Bias in estimating the overall log-odds-ratio, *θ*, by ${\hat{\theta }}_{MP}$ for $p_{C}=.1,\;\sigma^{2}=0.4$ (top three rows); *p_C_* = .4, $\sigma^{2}=0.4$ (bottom row), and constant sample sizes n=40,100. Top two rows: *θ* = 0; bottom two rows: θ=1.5. The data-generation mechanisms are FIM1 (circle), FIM2 (triangle), RIM1 (plus), RIM2 (cross), and URIM1 (diamond). Light gray line at 0.

The first three rows of Figure 5 illustrate the behavior when $p_{C}=.1$. The traces for FIM2 and RIM2 form one group, in which the bias does not vary with $\tau^{2}$; and those for FIM1, RIM1, and URIM1 form a second group, in which the bias increases with $\tau^{2}$. Under FIM2 and RIM2 ${\hat{\theta }}_{MP}$ is essentially unbiased when *θ* = 0 (as in the first row of Figure 5); but when θ>0, its bias is roughly −0.05 when *n *=* *40 (as in the third row of Figure 5), decreasing to nearly 0 when *n *=* *100. As *n* increases, the traces for FIM1, RIM1, and URIM1 flatten and also approach 0.

The fourth row of Figure 5 illustrates a situation, when $p_{C}=.4$, in which, for *K *=* *30, ${\hat{\theta }}_{MP}$ is nearly unbiased under FIM2 and RIM2 and has some negative bias under FIM1, RIM1, and URIM1, particularly when $\tau^{2}$ is larger. Other such situations involve *θ* = 0 or, mainly, *θ* = 2. Ordinarily, however, ${\hat{\theta }}_{MP}$ is essentially unbiased under all five data-generation mechanisms.

### 6.4 Bias of the estimators of θ in the FIM2 and RIM2 GLMMs

**Figure 6.**
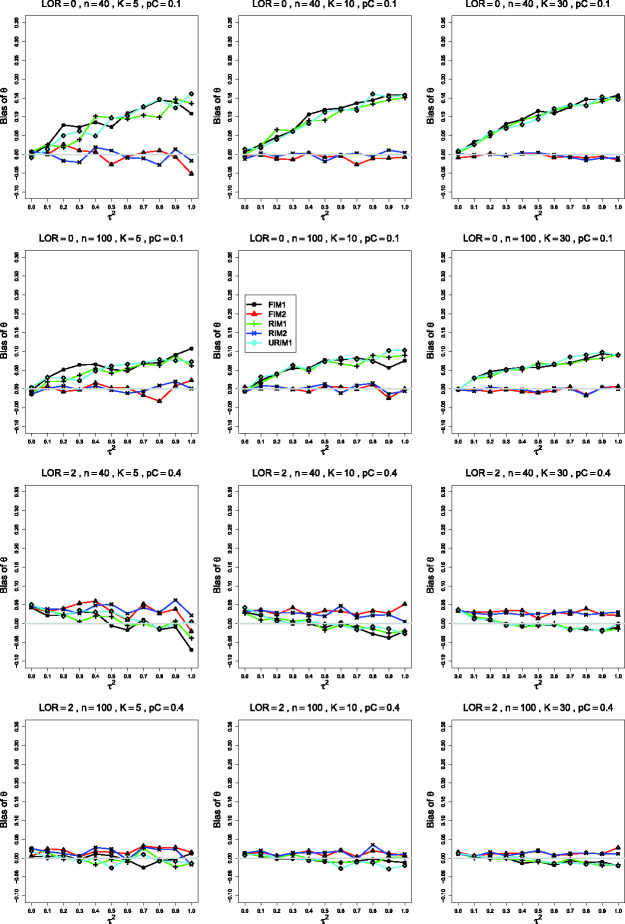
Bias of the estimator of the overall log-odds-ratio, *θ*, in the FIM2 GLMM when $\sigma^{2}=0.4$, constant sample sizes n=40,100, and $p_{C}=.1$ and *θ* = 0 (top two rows) or $p_{C}=.4$ and *θ* = 2 (bottom two rows). The data-generation mechanisms are FIM1 (circle), FIM2 (triangle), RIM1 (plus), RIM2 (cross), and URIM1 (diamond). Light gray line at 0.

**Figure 7.**
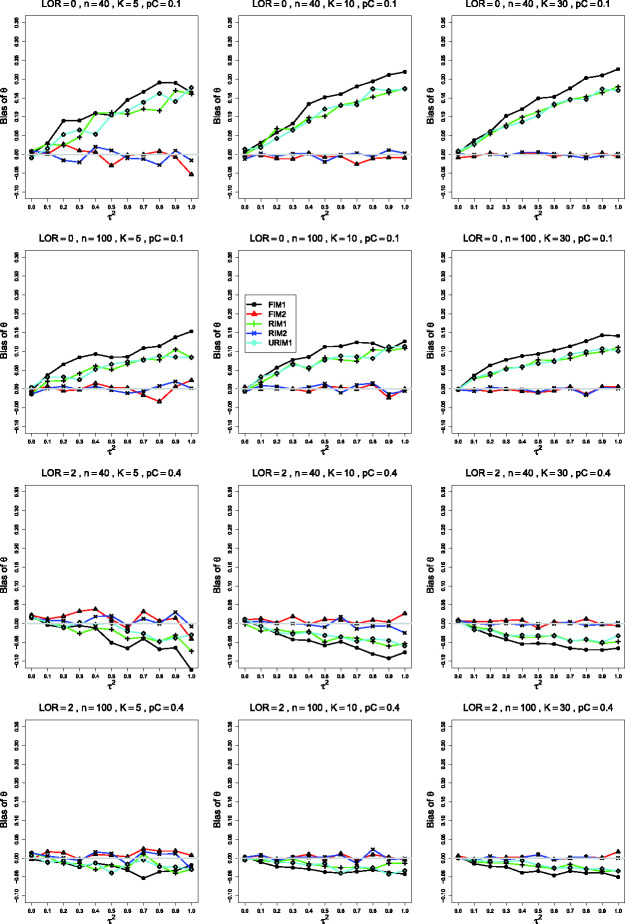
Bias of the estimator of the overall log-odds-ratio, *θ*, in the RIM2 GLMM when $\sigma^{2}=0.4$, constant sample sizes n=40,100, and $p_{C}=.1$ and *θ* = 0 (top two rows) or $p_{C}=.4$ and *θ* = 2 (bottom two rows). The data-generation mechanisms are FIM1 (circle), FIM2 (triangle), RIM1 (plus), RIM2 (cross), and URIM1 (diamond). Light gray line at 0.

For the bias of ${\hat{\theta }}_{\mathrm{FIM}2}$ and ${\hat{\theta }}_{\mathrm{RIM}2}$, the patterns of the traces in Figures 6 and 7 strongly resemble those for ${\hat{\theta }}_{MP}$. When $p_{C}=.1$, both estimators are essentially unbiased under FIM2 and RIM2, except for bias of + 0.01 to + 0.03 in ${\hat{\theta }}_{\mathrm{FIM}2}$ when θ≥1 and *n *=* *40. The behavior of the other group differs more clearly between ${\hat{\theta }}_{\mathrm{FIM}2}$ and ${\hat{\theta }}_{\mathrm{RIM}2}$: when *n *=* *40 and *n *=* *100, ${\hat{\theta }}_{\mathrm{RIM}2}$ usually has greater bias under FIM1 than under RIM1 or URIM1. (The plots for ${\hat{\theta }}_{MP}$ show a suggestion of this behavior.)

When $p_{C}=.4$, both ${\hat{\theta }}_{\mathrm{FIM}2}$ and ${\hat{\theta }}_{\mathrm{RIM}2}$ are usually unbiased under all five data-generation mechanisms. The exceptions arise mainly when *n *=* *40 (especially when *K *=* *30). When *θ* = 0, the traces for FIM1, RIM1, and URIM1 rise to around 0.02; when θ=1.5 or *θ* = 2, those traces drop to around −0.02 or lower.

### *6.5 Bias of the SSW estimator of* θ

**Figure 8.**
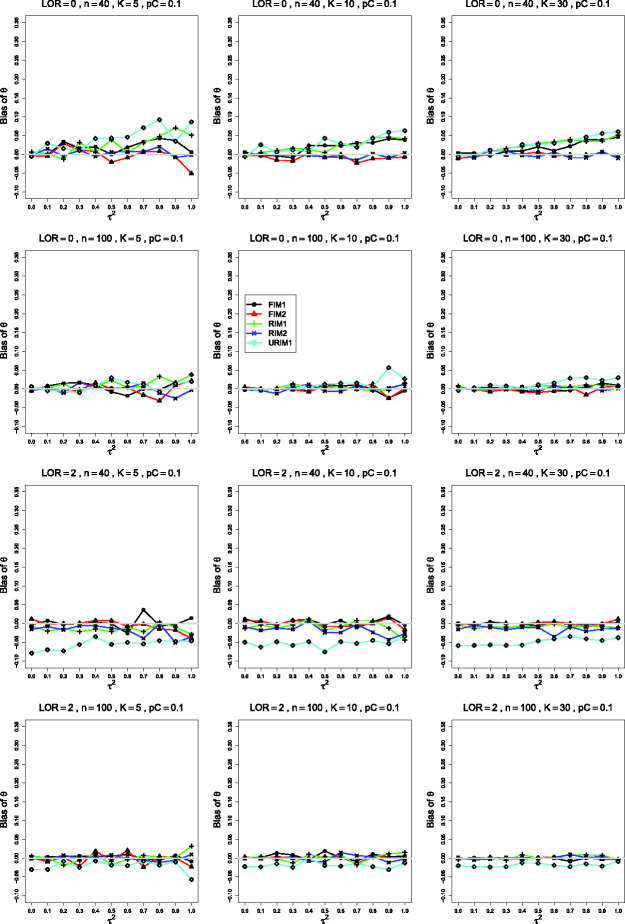
Bias of the SSW estimator of the overall log-odds-ratio, *θ*, for $p_{C}=.1$, $\sigma^{2}=0.4$, and constant sample sizes n=40;100. Top two rows, *θ* = 0; bottom two rows, *θ* = 2. The data-generation mechanisms are FIM1 (circle), FIM2 (triangle), RIM1 (plus), RIM2 (cross), and URIM1 (diamond). Light gray line at 0.

Only a few situations show bias in ${\hat{\theta }}_{\mathrm{SSW}}$. Those involve $p_{C}=.1$. When *θ* = 0, *n *=* *40, and *K *=* *10 and 30, the traces for FIM1, RIM1, and URIM1 are positive, rising to around 0.05 as $\tau^{2}$ increases to 1 (first row of Figure 8).

A different pattern arises when *θ* = 2 and $\sigma^{2}=0.4$; the trace for URIM1 is low, around −0.05 when *n *=* *40 (and *K *=* *5, 10, and 30) and around −0.02 when *n *=* *100 and *K *=* *30, shown in the third and fourth rows of Figure 8.

An explanation for this bias is that URIM1 may quite often produce extremely low or extremely high probabilities, as shown in Table 4. These probabilities may be even more extreme when $\tau^{2}$ is large. Then the relevant binomial distributions produce more zero or *n* values. Adding 0.5 to these data introduces the observed biases. This does not happen when $\sigma^{2}=0.1$ because then the probabilities are far enough from 0 and 1.

**Table 4.** Lower and upper bounds for *p_iC_* (*p_CL_* and *p_CU_*) and for *p_iT_* (*p_TL_* and *p_TU_*) values under URIM1.

*p_C_*	$\sigma^{2}$	*p_CL_*	*p_CU_*	*p_TL_*	*p_TU_*
.1	0.1	.0507	.1493	.2830	.5646
.1	0.4	.0014	.1986	.0103	.6468
.4	0.1	.2685	.5315	.7306	.8934
.4	0.4	.1371	.6629	.5400	.9356

Note: Intervals of *p_T_* are given for *b *=* *0 and *θ* = 2.

### *6.6 Coverage of the confidence interval for* θ *centered at* ${\hat{\theta }}_{MP}$

The 95% confidence interval for *θ* centered at ${\hat{\theta }}_{MP}$ uses normal critical values. The coverage of the confidence intervals based on the other IV-weighted estimators of *θ* has similar patterns.

With few exceptions, the patterns of the traces for coverage of the confidence interval based on ${\hat{\theta }}_{MP}$ are similar for $p_{C}=.1$ and $p_{C}=.4$. When *K *=* *5, all five start together above .95 at $\tau^{2}=0$. For $\tau^{2}\geq 0.1$ they decrease and then level off below .95 (as illustrated in Figure S1 in the Supplementary Material). As *K* increases, that level approaches .95, but increasing *n* has the opposite effect, producing coverage like that shown in the second row of Figure S1. Exceptions occur when *θ* = 0 and 0.5, $p_{C}=.1$, *n *=* *40, and *K *=* *10 and 30. Beyond a certain $\tau^{2}$ the traces separate into two groups; FIM2 and RIM2 level off around .95, and FIM1, RIM1, and URIM1 continue to decrease. Other, similar exceptions occur when *θ* = 0, $p_{C}=.1$, *n *=* *100, and *K *=* *30 and perhaps when *θ* = 2, $p_{C}=.4,\;\sigma^{2}=0.1$, *n *=* *40, and *K *=* *30.

### *6.7 Coverage of the confidence intervals for* θ *from the FIM2 and RIM2 GLMMs*

The coverage of the 95% confidence interval accompanying ${\hat{\theta }}_{\mathrm{FIM}2}$ generally resembles that of the confidence interval based on ${\hat{\theta }}_{MP}$ (compare Figure S2 and Figure S1). The main difference is that for all values of *θ*, the traces in the plot for *n *=* *40 and *K *=* *30 separate into the two groups (as illustrated in the first row of Figure S2).

The coverage of the confidence interval accompanying ${\hat{\theta }}_{\mathrm{RIM}2}$ has a surprising feature: When $p_{C}=.1$ and *n *=* *40, the traces for the five data-generation mechanisms often differ substantially (as in the first and third rows of Figure S3). Coverage may be close to .95 when $\tau^{2}=0$, but it can decline to .60 and below when $\tau^{2}=1$. Coverage under FIM2 generally exceeds .90, and it improves as *θ* increases. When $p_{C}=.4$ or n≥100, coverage of *θ* is similar to that from the FIM2 GLMM.

### *6.8 Coverage of the confidence interval centered at the SSW estimator of* θ

In all situations in our simulations, the traces for the coverage of the confidence interval centered at ${\hat{\theta }}_{\mathrm{SSW}}$ follow the null pattern. This favorable result makes it easy to summarize the coverage itself.

Coverage of the SSW interval exceeds .95 for small values of $\tau^{2}$. When $p_{C}=.1$, *n *=* *40, and *K *=* *5, coverage is still too high at $\tau^{2}=1$ (first row of Figure S4); this excess decreases somewhat when $p_{C}=.4$ (third row of Figure S4). It decreases when *K *=* *10, and coverage is close to nominal when *K *=* *30. Coverage approaches nominal for lower values of $\tau^{2}$ as the sample size increases. For *n *=* *1000, coverage is above nominal only at $\tau^{2}=0$ (second and fourth rows of Figure S4). Coverage does not depend on $\sigma^{2}$ or *θ*.

### 6.9 Summary

Our simulations explored two main components of design: the data-generation mechanism and the distribution of study-level sample sizes. As we mentioned earlier, the second of these had essentially no impact on bias of estimators of $\tau^{2}$, bias of estimators of *θ*, or coverage of confidence intervals for *θ*.

The five data-generation mechanisms often produced different results for at least one of those measures of performance. In the most frequent pattern, FIM2 and RIM2 yield similar results, and FIM1, RIM1, and URIM1 also yield results that are similar but different from those of FIM2 and RIM2. In some situations, URIM1 stands apart (e.g., for the bias of ${\hat{\tau }}_{MP}^{2}$ and the bias of ${\hat{\theta }}_{\mathrm{SSW}}$), and so does FIM1 (for the bias of ${\hat{\tau }}_{\mathrm{RIM}2}^{2}$ and the bias of ${\hat{\theta }}_{\mathrm{RIM}2}$). For *K *=* *30 Figure S3 shows a particularly unusual pattern, in which the traces for the five data-generation mechanisms are mostly separate.

In summary, except for the coverage of the SSW confidence interval and, in most situations, the bias of ${\hat{\theta }}_{\mathrm{SSW}}$, the choice of data-generation mechanism affects the results. These differences can complicate the process of integrating results from separate simulation studies.

## 7 Discussion

With the advent of powerful computers, the typical methodology paper in applied statistics has a standard structure. It proposes a new method, sometimes but not necessarily provides a mathematical derivation of its properties, and then uses simulation to demonstrate, usually successfully, that the new method is superior to previous methods.

Using methods for meta-analysis of odds ratios as an example, we aimed to compare various ways of generating data in simulations. In the literature, we identified five methods of generating odds ratios. We combined them with three methods of generating sample sizes, and we derived the statistical properties of inverse–variance–weighted estimators of the overall log-odds-ratio, *θ*, under these methods of data generation. In particular, we derived, to order 1/n, the biases and the variances of the inverse–variance–weighted estimators of *θ*.

We simulated data from the combinations of data-generation mechanism and sample-sizes method, and we compared the resulting estimates of the performance in estimating $\tau^{2}$ and *θ* of four methods of meta-analysis: inverse–variance weighting (represented by the Mandel-Paule method), the FIM2 and RIM2 GLMMs, and SSW (for *θ* only). Our results show that the properties of various methods and the recommendations on their use greatly depend on the data-generation mechanism.

Our theoretical derivations showed that, under FIM1/RIM1/URIM1, the IV-weighted estimators of *θ* should have positive bias for small values of $p_{T}<1/2$ and negative bias for $p_{T}>1/2$. On the other hand, under FIM2/RIM2 these estimators should be approximately unbiased when *θ* = 0. Our simulations (Figure 5) confirmed these findings.

Importantly, results of our simulations also show very similar behavior for the FIM2 and RIM2 GLMM estimators of *θ* (Figures 6 and 7). This finding is not very astonishing. Regardless of the hype that concerns use of GLMMs in meta-analysis, GLMs (and GLMMs) are asymptotic methods. The maximum-likelihood equations used in GLMs for binary data (Section 4.4 in McCullagh and Nelder^27^) are weighted-least-squares equations with inverse–variance weights. For this reason, the GLMMs result in quite considerable biases in meta-analysis of odds-ratios, as demonstrated by our simulations and by Bakbergenuly and Kulinskaya.^5^

The SSW estimator of *θ* had considerably less bias, but even for this estimator the data-generation mechanism mattered, as URIM1 produced more-biased results (Figure 8).

Differences in the behavior of moment-based estimators of $\tau^{2}$ such as ${\hat{\tau }}_{MP}^{2}$ under various data-generation mechanisms (Figures 1 and 2) have the same explanation as those for estimators of *θ*. These estimators are derived from the *Q* statistic, which is affected by the correlation between the effects and the weights.

Even though wider, *t*-based confidence intervals^17,28,29^ would somewhat improve coverage of *θ*, differences in coverage are due perhaps more to the centering of the intervals at very biased estimators. These biases are so large that they obscured the results of inflated variance in RIM methods. We also did not observe differences associated with random generation of sample sizes, perhaps because we used relatively tight intervals for them.

Finally, an interesting question is whether particular estimation methods work better when the data are generated exactly from the assumed model. Counterintuitively, the answer is no. In the majority of our simulations, generation under FIM2/RIM2 resulted in better estimation by all methods. But the RIM2 GLMM produced confidence intervals for *θ* that had much better coverage when the data were generated under FIM2, and really bad coverage otherwise.

What method(s) of meta-analysis should be used in practice, where we can never be certain of the true data-generating mechanism? In estimating *θ*, SSW provides the lowest biases and coverage that is correct but rather conservative and appears to be robust to the data-generation mechanism. This advantage will be shared by other methods that use constant weights.

As a more robust alternative in the two-stage random-effects model, Henmi and Copas^30^ and, independently, Stanley and Doucouliagos^31^ use an inverse–variance–weighted fixed-effect (FE) estimator as the center of the CI for *θ*. Our results show that the FE estimator of *θ* is also biased and will be affected by the simulation method.

Our findings are not surprising when put in a wider context. In pursuit of the effect of interest, we often neglect nuisance parameters that are sometimes only implicitly present in our models. However, when the sufficient statistics include these nuisance parameters, their distribution matters. Different distribution assumptions for the nuisance parameters should and do result in different properties of the estimators of interest. This influence directly parallels the effects of choice of prior distribution on the properties of the increasingly common Bayesian variants of the two-stage and GLM meta-analytic methods.^8,32,33^ One solution may be to try to develop minimax procedures that would minimize possible biases. Another solution is the use of procedures that are robust to a wide class of distributions for nuisance parameters.

We demonstrated substantial effects of data-generating mechanisms on the inference in meta-analysis of odds-ratios. These complications are not restricted to binary data, and they make it difficult to rely on any single simulation in choosing methods. Careful, resourceful effort may lead to a battery of designs that, collectively, approximates the mechanisms underlying the data in actual meta-analyses. In any event, simulations should be designed with the awareness of the possible effects of design choices, and quite a few recommendations may need to be revised.

## Supplemental Material

sj-pdf-1-smm-10.1177_09622802211013065 - Supplemental material for Exploring consequences of simulation design for apparent performance of methods of meta-analysis Click here for additional data file. Supplemental material, sj-pdf-1-smm-10.1177_09622802211013065 for Exploring consequences of simulation design for apparent performance of methods of meta-analysis by Elena Kulinskaya, David C. Hoaglin and Ilyas Bakbergenuly in Statistical Methods in Medical Research

## Appendix 1. Derivations for equations (12) and (13)

This appendix gives derivations for equations (12) and (13). From equations (3) and (4)

$${\hat{\theta }}_{i}=\text{logit}({\hat{p}}_{iT})-\text{logit}({\hat{p}}_{iC})$$

and

$$\hat{\text{Var}}({\hat{\theta }}_{i})={\hat{v}}_{i}^{2}=\frac{1}{n_{iT}}\left(\frac{1}{{\hat{p}}_{iT}}+\frac{1}{1-{\hat{p}}_{iT}}\right)+\frac{1}{n_{iC}}\left(\frac{1}{{\hat{p}}_{iC}}+\frac{1}{1-{\hat{p}}_{iC}}\right)$$

In Section 3.1, we rewrote equation (10) as

$$\text{Cov}({\hat{\theta }}_{i},{\hat{v}}_{i}^{2})=\text{E}[\text{Cov}({\hat{\theta }}_{i},{\hat{v}}_{i}^{2}|p_{iC},\;p_{iT})]+\text{Cov}(\theta_{i},v_{i}^{2})$$

We first calculate $\text{Cov}({\hat{\theta }}_{i},{\hat{v}}_{i}^{2}|p_{iC},\;p_{iT})$. Since ${\hat{p}}_{iT}$ and ${\hat{p}}_{iC}$ are conditionally independent, we expand their terms of ${\hat{\theta }}_{i}$ and ${\hat{v}}_{i}^{2}$ separately

$$\text{logit}(\hat{p})=\text{logit}(p)+(\hat{p}-p)\left(\frac{1}{p}+\frac{1}{1-p}\right)-\frac{{\left(\hat{p}-p\right)}^{2}}{2}\left(\frac{1}{p^{2}}-\frac{1}{{\left(1-p\right)}^{2}}\right)+\cdots$$

and (omitting the 1/n)

$$\frac{1}{\hat{p}}+\frac{1}{1-\hat{p}}=\frac{1}{p}+\frac{1}{1-p}+(\hat{p}-p)\left(\frac{1}{{\left(1-p\right)}^{2}}-\frac{1}{p^{2}}\right)+{\left(\hat{p}-p\right)}^{2}\left(\frac{1}{{\left(1-p\right)}^{3}}+\frac{1}{p^{3}}\right)$$

For each of T and C, the conditional covariance of these two terms has the form

$$\text{Cov}(a(\hat{p}-p)+b{\left(\hat{p}-p\right)}^{2},c(\hat{p}-p)+d{\left(\hat{p}-p\right)}^{2}|p)=ac\text{Var}(\hat{p}|p)+O(1/n^{2})$$

because the variance of the binomial distribution is of order 1/n and the higher central moments are at most $O(1/n^{2})$. For $a={\left[p(1-p)\right]}^{-1}$ and $c=(p^{2}-{\left(1-p\right)}^{2})/[p^{2}{\left(1-p\right)}^{2}]=(2p-1)/[p^{2}{\left(1-p\right)}^{2}]$

$$\text{Cov}\left(\text{logit}(\hat{p}),\frac{1}{\hat{p}}+\frac{1}{1-\hat{p}}|p\right)=\frac{1}{n}\left[\frac{2p-1}{p^{2}{\left(1-p\right)}^{2}}\right]$$

Combining the results for T and C (and restoring the 1/n) yields

$$\text{Cov}\left({\hat{\theta }}_{i},{\hat{v}}_{i}^{2}|p_{iC},\;p_{iT}\right)=\frac{1}{n_{iT}^{2}}\left[\frac{2p_{iT}-1}{p_{iT}^{2}{\left(1-p_{iT}\right)}^{2}}\right]-\frac{1}{n_{iC}^{2}}\left[\frac{2p_{iC}-1}{p_{iC}^{2}{\left(1-p_{iC}\right)}^{2}}\right]$$

which is only $O(1/n^{2})$, so we do not need to calculate its expectation.

Next we calculate the second term of equation (10), $\text{Cov}(\theta_{i},v_{i}^{2})$. Under the RIM, equation (7)

(16) $$\begin{array}{l} p_{iT}^{-1}=1+\exp(-(\alpha +u_{i}+\theta +(1-c)b_{i})),\\ (1-p_{iT}{)}^{-1}=1+\exp(\alpha +u_{i}+\theta +(1-c)b_{i}),\\ p_{iC}^{-1}=1+\exp(-(\alpha +u_{i}-cb_{i})),\\ \left(1-p_{iC}{)}^{-1}=1+\exp(\alpha +u_{i}-cb_{i}\right) \end{array}$$

Substituting these expressions in equation (4) yields

$$\begin{array}{l} v_{i}^{2}=n_{iC}^{-1}\left[e^{-(\alpha +u_{i}-cb_{i})}+2+e^{\alpha +u_{i}-cb_{i}}\right]+n_{iT}^{-1}\left[e^{-(\alpha +u_{i}+\theta +(1-c)b_{i})}+2+e^{\alpha +u_{i}+\theta +(1-c)b_{i}}\right] \end{array}$$

Because $\theta_{i}=\theta +b_{i}$, to complete the calculation, we need covariances of the form $\text{Cov}(b,e^{dx})$. As $e^{dx}=e^{dx_{0}}+d(x-x_{0})e^{dx_{0}}+{\left(x-x_{0}\right)}^{2}d^{2}e^{dx_{0}}/2+\cdots$, where $x_{0}=\text{E}(x)$, we have

(17) $$\text{Cov}(b,e^{dx})=d\text{Cov}(b,x)e^{dx_{0}}+\text{Cov}(b,{\left(x-x_{0}\right)}^{2})d^{2}e^{dx_{0}}/2+\cdots$$

For simplicity, we assume that *u_i_* and *b_i_* are independent. Then, to order 1/n

(18) $$\text{Cov}(\theta_{i},v_{i}^{2})=n_{iC}^{-1}c\tau^{2}[e^{-\alpha }-e^{\alpha }]-n_{iT}^{-1}(1-c)\tau^{2}[e^{-\alpha -\theta }-e^{\alpha +\theta }]$$

In particular, when *c *=* *1/2, *θ* = 0 and *n_iT_* = *n_iC_*, $\text{Cov}(\theta_{i},v_{i}^{2})=0$. Defining $p_{C}=\text{expit}(\alpha )$ and $p_{T}=\text{expit}(\alpha +\theta )$ yields $e^{-\alpha }-e^{\alpha }=p_{C}^{-1}-{\left(1-p_{C}\right)}^{-1}$ and $e^{-\alpha -\theta }-e^{\alpha +\theta }=p_{T}^{-1}-{\left(1-p_{T}\right)}^{-1}$ and the equivalent expression, equation (12)

$$\text{Cov}(\theta_{i},v_{i}^{2})=\frac{c\tau^{2}}{n_{iC}}\left[\frac{1-2p_{C}}{p_{C}(1-p_{C})}\right]-\frac{(1-c)\tau^{2}}{n_{iT}}\left[\frac{1-2p_{T}}{p_{T}(1-p_{T})}\right]$$

Similarly, from equation (11), $\text{Var}({\hat{\theta }}_{i})=\text{E}(v_{i}^{2})+\tau^{2}$. To calculate $\text{E}(v_{i}^{2})$, we need expansions in two variables, *u_i_* and *b_i_*. We omit the grubby details, observe that $e^{-\alpha }+e^{\alpha }={\left[p_{C}(1-p_{C})\right]}^{-1}-2$ and $e^{-\alpha -\theta }+e^{\alpha +\theta }={\left[p_{T}(1-p_{T})\right]}^{-1}-2$, and express the full variance of ${\hat{\theta }}_{i}$ in terms of *p_C_* and *p_T_*

(19) $$\begin{array}{l} \text{Var}({\hat{\theta }}_{i})=[n_{iT}p_{T}(1-p_{T}){]}^{-1}+[n_{iC}p_{C}(1-p_{C}){]}^{-1}+\tau^{2}\\ \;+\left(\sigma^{2}+{\left(1-c\right)}^{2}\tau^{2}+2(1-c)\rho \sigma \tau \right)[2n_{iT}{]}^{-1}\left({\left[p_{T}(1-p_{T})\right]}^{-1}-2\right)\\ \;+\left(\sigma^{2}+c^{2}\tau^{2}-2c\rho \sigma \tau \right)[2n_{iC}{]}^{-1}\left({\left[p_{C}(1-p_{C})\right]}^{-1}-2\right) \end{array}$$

This is equation (13).

## Appendix 2. Arbitrary distribution for p_c_

To calculate $\text{Var}({\hat{\theta }}_{i})$ for an arbitrary distribution of *p_iC_* but assuming that the *p_iC_* and the normal random effects *b_i_* are independent, we proceed as in equation (19) to obtain (for *c *=* *0 and substituting $p_{C}^{0}=\text{expit}(\text{E}(\text{logit}(p_{iC}))),\;p_{T}^{0}=\text{expit}(\text{E}(\text{logit}(p_{iC}))+\theta )$ and $\text{Var}(\text{logit}(p_{iC}))$ for *p_C_*, *p_T_* and $\sigma^{2}$, respectively)

$$\begin{array}{l} \text{Var}({\hat{\theta }}_{i})=[n_{iT}p_{T}^{0}(1-p_{T}^{0}){]}^{-1}+[n_{iC}p_{C}^{0}(1-p_{C}^{0}){]}^{-1}+\tau^{2}\\ \;+\left(\text{Var}(\text{logit}(p_{iC}))+\tau^{2}\right)[2n_{iT}{]}^{-1}\left({\left[p_{T}^{0}(1-p_{T}^{0})\right]}^{-1}-2\right)\\ \;+\left(\text{Var}(\text{logit}(p_{iC}))\right)[2n_{iC}{]}^{-1}\left({\left[p_{C}^{0}(1-p_{C}^{0})\right]}^{-1}-2\right) \end{array}$$

$\text{Cov}(\theta_{i},v_{i}^{2})$ is the same as in equation (12) with *c *=* *0 and $p_{T}^{0}=\text{expit}(\text{E}(\text{logit}(p_{iC}))+\theta )$, i.e.

(20) $$\text{Cov}(\theta_{i},v_{i}^{2})=-\frac{\tau^{2}}{n_{iT}}\left[\frac{1-2p_{T}^{0}}{p_{T}^{0}(1-p_{T}^{0})}\right]$$

This should produce substantial positive bias in ${\hat{w}}_{i}{\hat{\theta }}_{i}$ for small $p_{T}^{0}<1/2$, and negative bias for $p_{T}^{0}>1/2$.

To evaluate the first two moments of $\text{logit}(p_{iC})$, we use the standard delta method, as in equation (8). Let the mean and the variance of the distribution of *p_iC_* be *p_C_* and $\sigma_{C}^{2}$. For h(x)=logit(x), the derivatives of h(·) at *p_C_* are

$$h^{\prime }(p_{C})=\frac{1}{p_{C}(1-p_{C})}\;\text{and}\;h^{\prime \prime }(p_{C})=-\left[\frac{1}{p_{C}^{2}}+\frac{1}{{\left(1-p_{C}\right)}^{2}}\right]$$

Hence

$$\text{E}(\text{logit}(p_{iC}))=\text{logit}(p_{C})-\left[\frac{1}{p_{C}^{2}}+\frac{1}{{\left(1-p_{C}\right)}^{2}}\right]\sigma_{C}^{2}/2$$

and

$$\text{Var}(\text{logit}(p_{iC}))=\frac{1}{p_{C}^{2}{\left(1-p_{C}\right)}^{2}}\sigma_{C}^{2}$$

The expected value decreases, and the variance increases, with $\sigma_{C}^{2}$, the variance of the distribution of *p_iC_*.

For a uniform distribution on an interval of width Δ centered at *p_C_*, $\sigma_{C}^{2}=\Delta^{2}/12$.
