# Supplementary material for: Exploring consequences of simulation design for apparent performance of methods of meta-analysis
Source: Stat Methods Med Res. 2021 Jun 10;30(7):1667–90. doi: 10.1177/09622802211013065 (PMC8411476; doi:10.1177/09622802211013065)
Supplement: sj-pdf-1-smm-10.1177_09622802211013065 - Supplemental material for Exploring consequences of simulation design for apparent performance of methods of meta-analysis [file sj-pdf-1-smm-10.1177_09622802211013065.pdf]

Supplemental Material for "Exploring Consequences of Simulation Design for Apparent Performance of Methods of Meta-analysis"

Additional Figures S1-S4 on Coverage of Overall Log-Odds-Ratio θ

Elena Kulinskaya, David C. Hoaglin and Ilyas Bakbergenuly

March 1, 2021

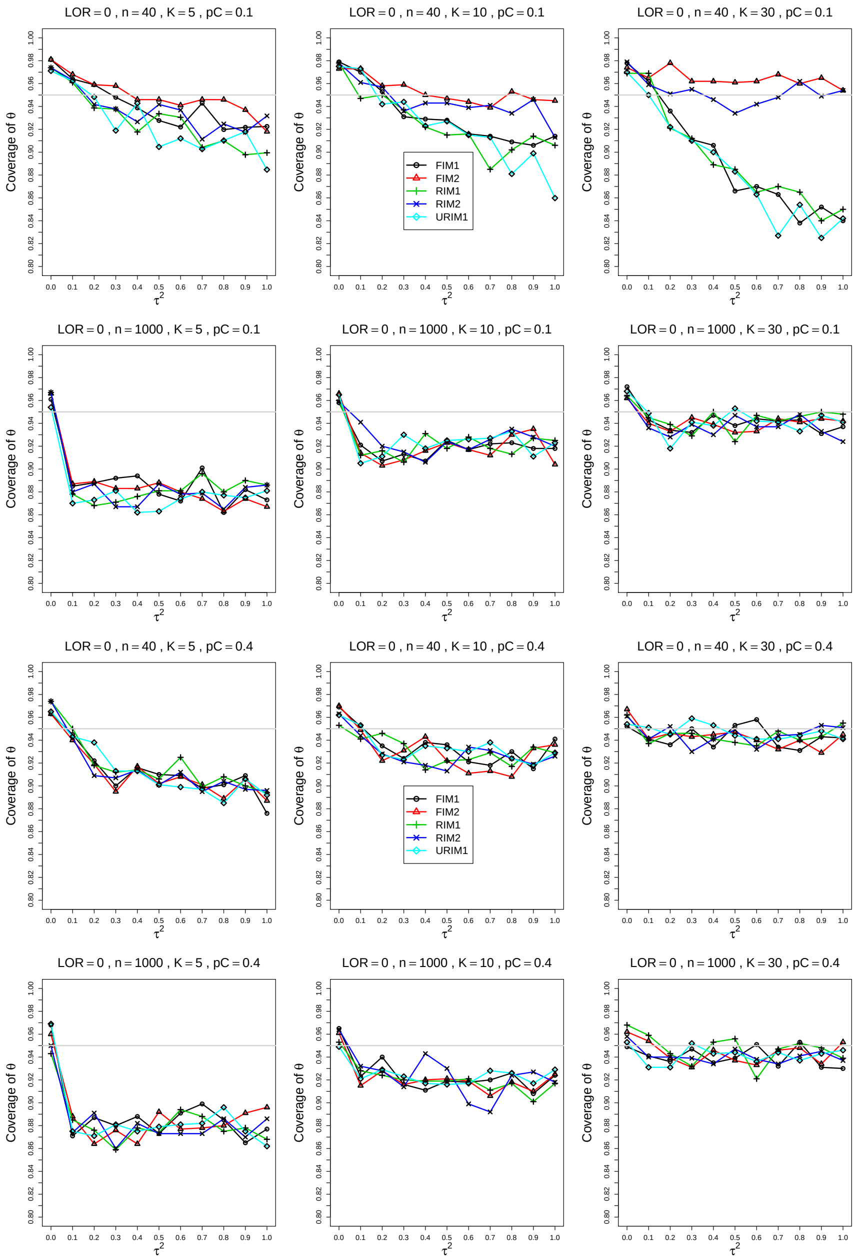

Figure S1: Coverage of the overall log-odds-ratio θ by a 95% normal confidence interval centered at $\hat{\theta}_{MP}$ for $\theta = 0$, $\sigma^2 = 0.4$, constant sample sizes $n = 40; 1000$, $p_C = .1$, (top 2 rows) or $p_C = .4$ (bottom two rows). The data-generation mechanisms are FIM1 (circle), FIM2 (triangle), RIM1 (plus), RIM2 (cross), and URIM1 (diamond). Light grey line at .95.

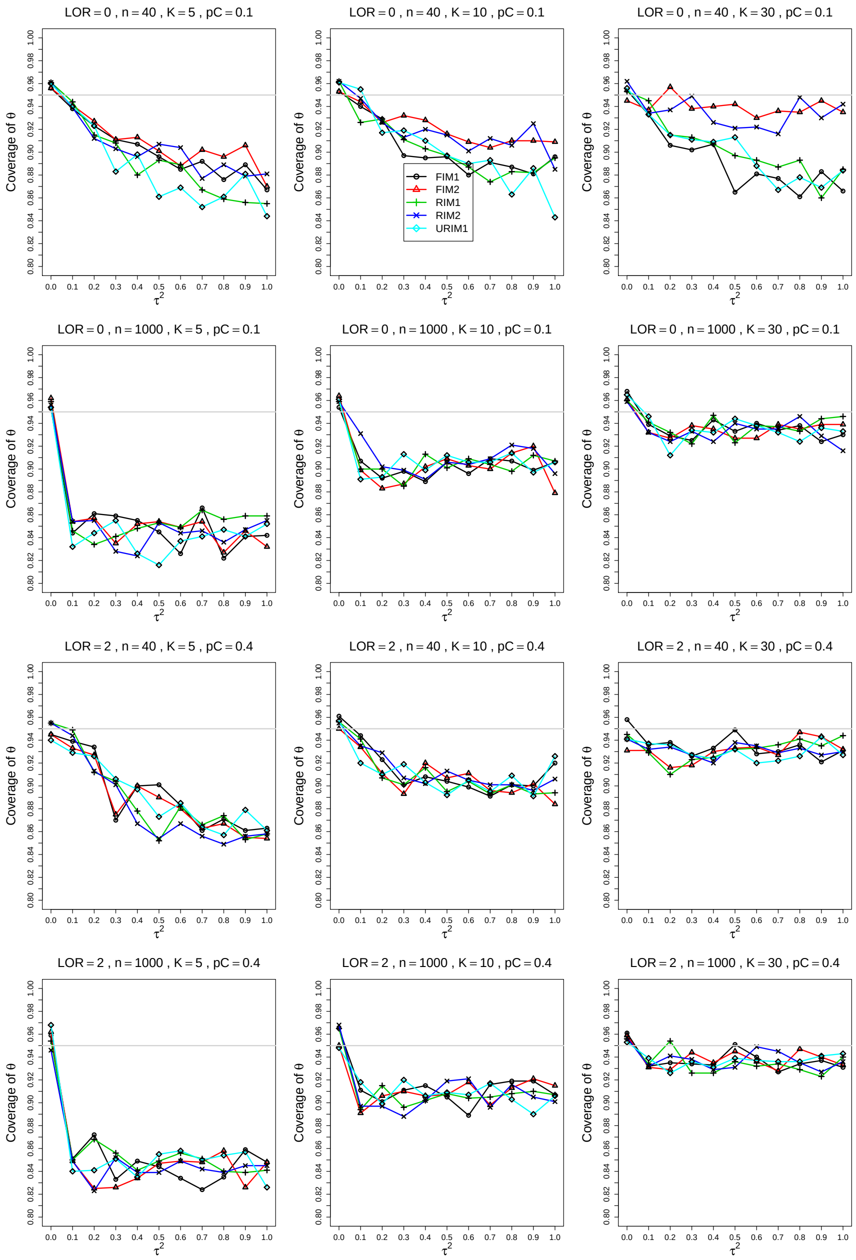

Figure S2: Coverage of the overall log-odds ratio θ by the 95% confidence interval from the FIM2 GLMM for $\sigma^2 = 0.4$; constant sample sizes $n = 40$, 1000; $p_{iC} = 0.1$, $\theta = 0$ (top 2 rows) or $p_{iC} = 0.4$, $\theta = 2$ (bottom two rows). The data-generation mechanisms are FIM1 (circle), FIM2 (triangle), RIM1 (plus), RIM2 (cross), and URIM1 (diamond). Light grey line at .95.

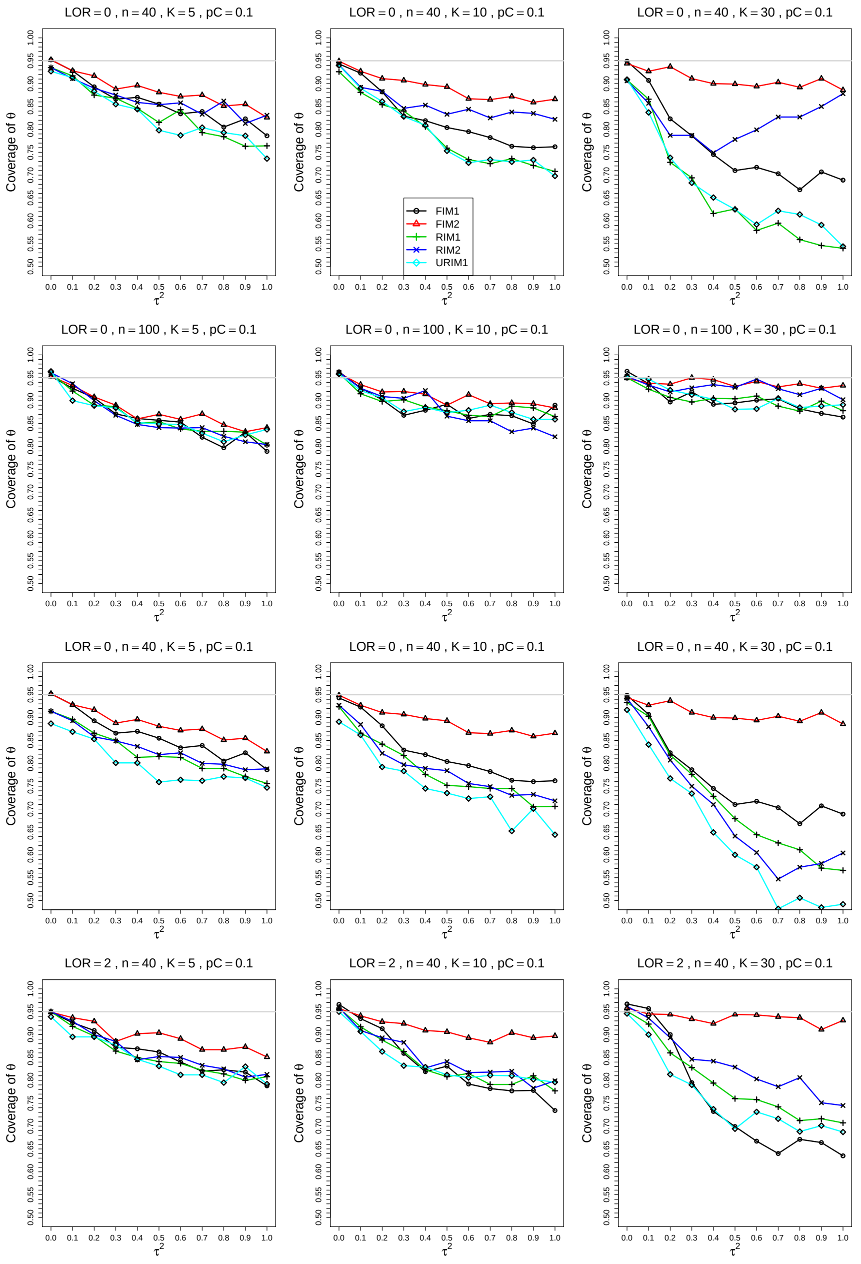

Figure S3: Coverage of the overall log-odds ratio θ by the 95% confidence interval from the RIM2 GLMM for $p_C = .1$, $\theta = 0$, $\sigma^2 = 0.1$ (top two rows), $\theta = 0$, $\sigma^2 = 0.4$ (third row) and $\theta = 2$, $\sigma^2 = 0.4$ (bottom row), constant sample sizes $n = 40;\ 1000$. The data-generation mechanisms are FIM1 (circle), FIM2 (triangle), RIM1 (plus), RIM2 (cross), and URIM1 (diamond). Light grey line at .95.

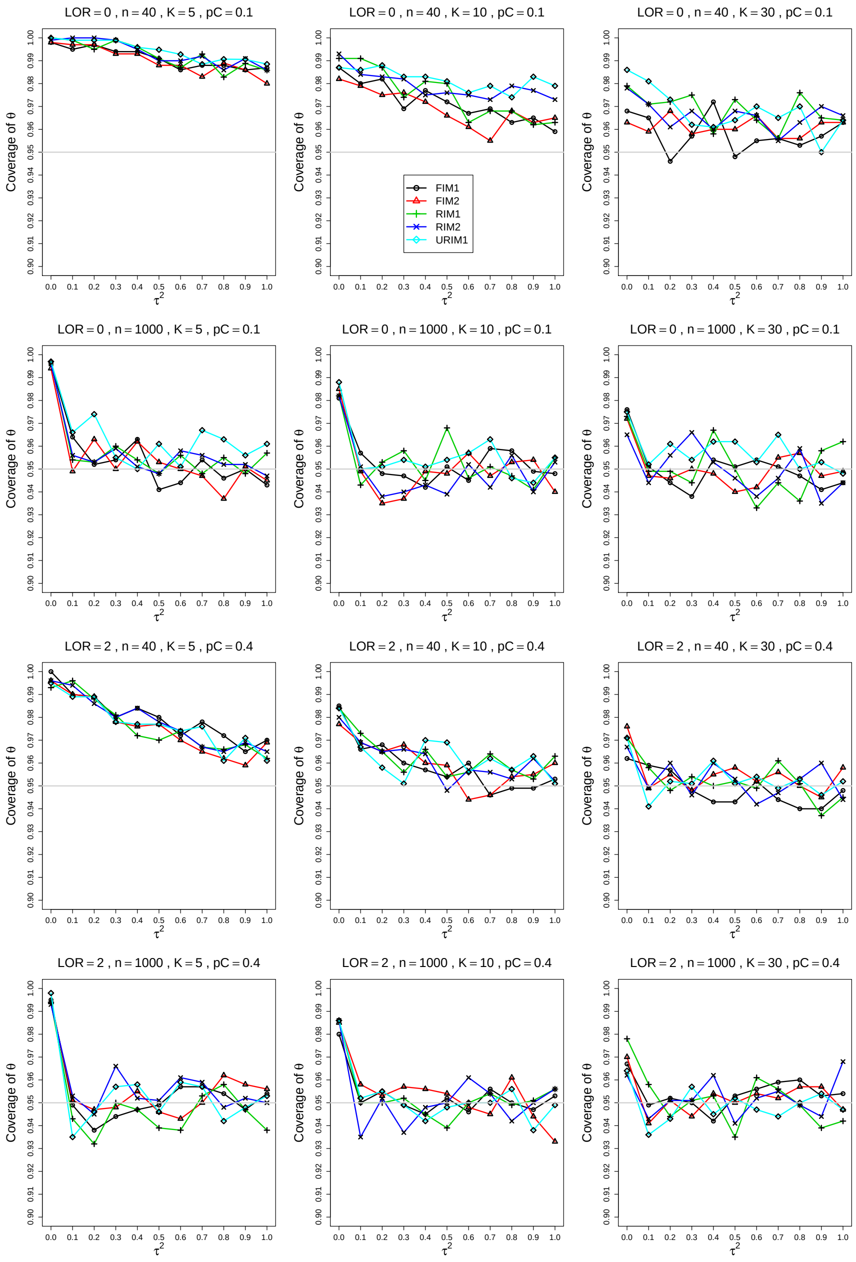

Figure S4: Coverage of the overall log-odds-ratio θ by the 95% t confidence interval centered at $\hat{\theta}_{SSW}$ for $\sigma^2 = 0.4$, constant sample sizes $n = 40; 1000$, $p_C = .1$, $\theta = 0$, (top 2 rows) or $p_C = .4$, $\theta = 2$ (bottom two rows). The data-generation mechanisms are FIM1 (circle), FIM2 (triangle), RIM1 (plus), RIM2 (cross), and URIM1 (diamond). Light grey line at .95.
